# Design, Synthesis, and Evaluation of New 2-Arylpropanoic Acid-l-Tryptophan Derivatives for Mitigating Cisplatin-Induced Nephrotoxicity

**DOI:** 10.3390/molecules30112400

**Published:** 2025-05-30

**Authors:** Ming Yuan, Huai Wang, Mingjun Yu, Sen Yao, Risheng Yao

**Affiliations:** 1 School of Food and Biological Engineering, Hefei University of Technology, Hefei 230601, China; psv1221@163.com (M.Y.); yuymj127@126.com (M.Y.); yaosentc@163.com (S.Y.); 2 School of Traditional Chinese Medicine, Bozhou University, Bozhou 236800, China

**Keywords:** cisplatin, nephrotoxicity, gastrin-releasing peptide receptor, PD176252, 2-arylpropanoic acid, anti-inflammatory

## Abstract

Cisplatin (CIS) is a widely used chemotherapeutic agent that is highly effective against various cancers. However, its clinical application is frequently limited by its substantial nephrotoxic side effects. The gastrin-releasing peptide receptor (GRPR), a critical regulator in inflammatory diseases, has been identified as a promising therapeutic target. Our previous studies have demonstrated that the GRPR antagonists PD176252 and RH-1402 can mitigate CIS-induced nephrotoxicity through anti-inflammatory mechanisms. Based on these findings, we designed and synthesized a series of 2-arylpropanoic acid-L-tryptophan derivatives to enhance the therapeutic effects. Among these compounds, **3m** exhibited superior renal protection by significantly improving mouse renal tubular epithelial cell (mRTEC) viability from 50.2 ± 2.6% to 80.5 ± 3.9%, surpassing PD176252 (70.8 ± 1.4%) and RH-1402 (73.9 ± 3.7%). Moreover, compound **3m** markedly reduced the expression of kidney injury molecule-1 (KIM-1) and inflammatory cytokines [Tumor Necrosis Factor-α (TNF-α), Interleukin-6 (IL-6), Monocyte Chemoattractant Protein-1 (MCP-1)]. Finally, molecular docking results revealed that **3m** exhibited a high binding affinity for GRPR. Computational predictions using SwissADME further indicated that **3m** possesses favorable drug-like properties, thereby supporting its potential as a promising candidate for mitigating CIS-induced nephrotoxicity.

## 1. Introduction

Cisplatin (CIS) is a widely used anticancer agent in the treatment of various malignancies, including cervical, ovarian, head and neck, prostate, testicular, and bladder cancers [1,2,3]. However, its prolonged use is frequently hindered by nephrotoxicity, a significant adverse effect observed in patients [4]. CIS-induced nephrotoxicity encompasses acute kidney injury (AKI), renal tubular dysfunction, hyperuricemia, hypomagnesemia, hypocalcemia, and chronic kidney disease [5]. It is estimated that approximately 30% of cancer patients receiving CIS therapy experience a substantial decline in kidney function, which negatively impacts their prognosis and overall health [6,7]. Despite the clinical relevance of CIS-induced nephrotoxicity, there are currently limited specific treatment options [8].

CIS predominantly accumulates within the renal tubules, causing more extensive damage to the kidneys compared to other organs. As a result, nephrotoxicity is regarded as the most severe adverse effect of CIS [9]. The pathophysiology of CIS-induced nephrotoxicity is complex, involving multiple mechanisms, including inflammation, oxidative stress, apoptosis, and mitochondrial dysfunction [10,11]. Inflammation plays a central role in this renal toxic response. CIS stimulates the release of pro-inflammatory cytokines, activates local inflammatory pathways, exacerbates renal cell damage, and may promote the development of kidney fibrosis [12,13]. CIS induces oxidative stress by generating reactive oxygen species (ROS), which impair kidney cells by damaging cellular membranes, proteins, lipids, and DNA, ultimately leading to cell death [14]. Additionally, CIS interacts with mitochondria, disrupting their function, reducing adenosine triphosphate (ATP) synthesis, and increasing mitochondrial membrane permeability, thereby causing mitochondrial damage and cell death [15]. Consequently, CIS-induced nephrotoxicity results in both the apoptosis and necrosis of renal cells, severely compromising kidney function.

The gastrin-releasing peptide (GRP) is a neuropeptide secreted by both neurons and endocrine cells, initially identified in the stomach of mammals and later detected in the brain and other tissues [16]. The GRP modulates various physiological processes by binding to its receptor, the gastrin-releasing peptide receptor (GRPR), and influences functions such as central nervous system regulation, gastrointestinal activity, tumor progression, immune cell modulation, and inflammatory responses [17,18,19,20]. The GRPR plays a pivotal role in renal injury induced by CIS. The GRP, in conjunction with the GRPR, participates in biological processes such as inflammation, oxidative stress, and apoptosis, thus exacerbating the nephrotoxicity associated with CIS. GRPR antagonists can inhibit several pro-inflammatory signaling pathways, prevent the secretion of pro-inflammatory cytokines, and reduce the infiltration of inflammatory cells, thereby mitigating renal inflammation and alleviating kidney damage [21]. Furthermore, GRPR antagonists can diminish oxidative stress, reduce ROS production, and prevent kidney cell death resulting from oxidative damage [22]. GRPR antagonists also suppress apoptosis-related signaling pathways, thereby protecting damaged renal cells [23]. In addition, numerous studies have highlighted the critical role of inflammation in CIS-induced nephrotoxicity. For instance, Tim-3, Rosiridin, and chitosan–ascorbate nanocapsules have been shown to effectively reduce CIS-induced nephrotoxicity by inhibiting renal inflammation [24,25,26].

Our previous research demonstrated that the small-molecule GRPR antagonist PD176252 alleviates CIS-induced nephrotoxicity, whereas RH-1402, a derivative designed based on PD176252, exhibits enhanced renal protective effects by reducing inflammation and mitigating kidney damage [21,27]. In the current study, we further optimized the structure of RH-1402 and synthesized a series of novel 2-arylpropanoic acid-L-tryptophan derivatives. Mouse renal tubular epithelial cells (mRTECs) are closely associated with the physiological functions of the kidneys and are widely used as a model for studying renal toxicity, particularly CIS-induced kidney injury [27]. Additionally, the cultivation of mRTECs is straightforward and easy to manage. Therefore, we selected mRTECs to assess the protective effects of the newly synthesized derivatives against CIS-induced cell death and their capacity to inhibit inflammatory responses.

## 2. Results and Discussion

### 2.1. Design and Synthesis of Compounds **3a**–**o**

#### 2.1.1. Design

**Figure 1:**
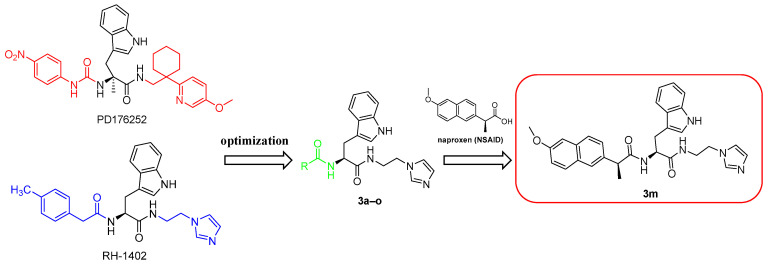
Design of target compounds **3a**–**o**.

Non-steroidal anti-inflammatory drugs (NSAIDs) are among the most commonly prescribed medications for the treatment of inflammatory diseases. The 2-arylpropanoic acid moiety is a crucial structural component in several NSAIDs, including ibuprofen, ketoprofen, and naproxen. Recent studies have indicated that incorporating the NSAID functional group may enhance the biological activity of specific compounds [28,29,30]. Given that 2-arylpropionic acid shares structural similarities with the *p*-tolylacetic acid group in RH-1402, this substitution may preserve some of the pharmacological benefits of the original compound while facilitating a simpler synthetic route. To further augment the renal protective properties, we replaced the *p*-tolylacetic acid group in RH-1402 with 2-arylpropionic acid, thereby yielding the target compound (Figure 1).

#### 2.1.2. Synthesis

**Scheme 1:**
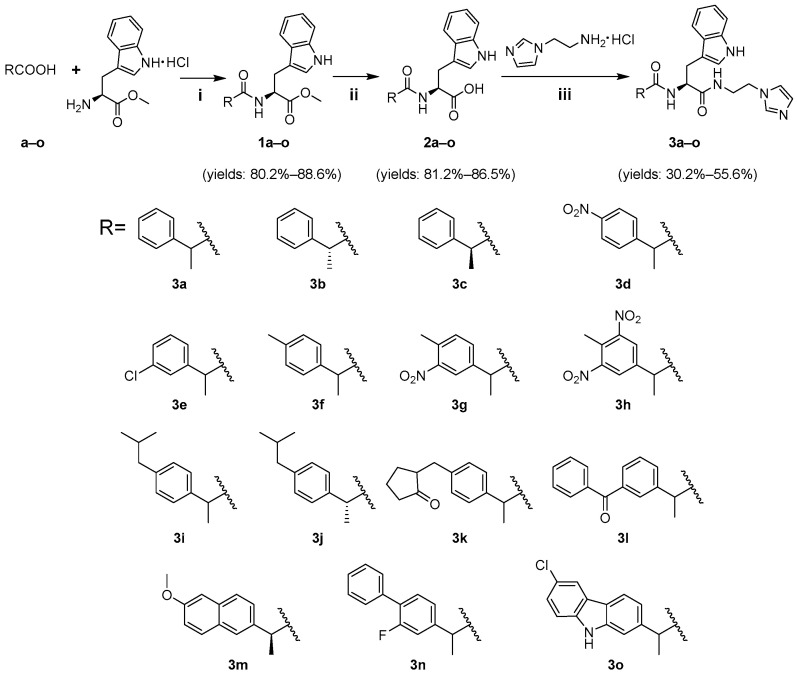
Synthesis of target compounds **3a**–**o**. Reaction conditions: (**i**) EDCI, HOBt, DIPEA, DCM, rt., 7–8 h; (**ii**) Na_2_CO_3_, methanol, 55 °C, 5–6 h; (**iii**) EDCI, HOBt, DIPEA, DCM, rt., 24 h.

Scheme 1 illustrates the synthetic pathway leading to the target compounds **3a**–**o**. Initially, intermediates **1a**–**o** were synthesized through the reaction of 2-arylpropanoic acid derivatives **a**–**o** with L-tryptophan methyl ester hydrochloride. Subsequent hydrolysis of these intermediates afforded compounds **2a**–**o**. Finally, the reaction of compounds **2a**–**o** with 2-(1*H*-imidazol-1-yl)ethan-1-amine resulted in the formation of the target compounds **3a**–**o**.

### 2.2. Biological Activity

#### 2.2.1. Compounds **3a**–**o** Mitigate CIS-Induced mRTEC Death

CIS-induced nephrotoxicity is primarily characterized by significant damage to renal tubular epithelial cells [31,32,33]. The CCK-8 assay was employed to evaluate the protective effects of compounds **3a**–**o** against CIS-induced cell death in mRTEC [31,34]. Cell viability was measured after treatment with CIS alone or in combination with varying concentrations of the target compounds.

**Figure 2:**
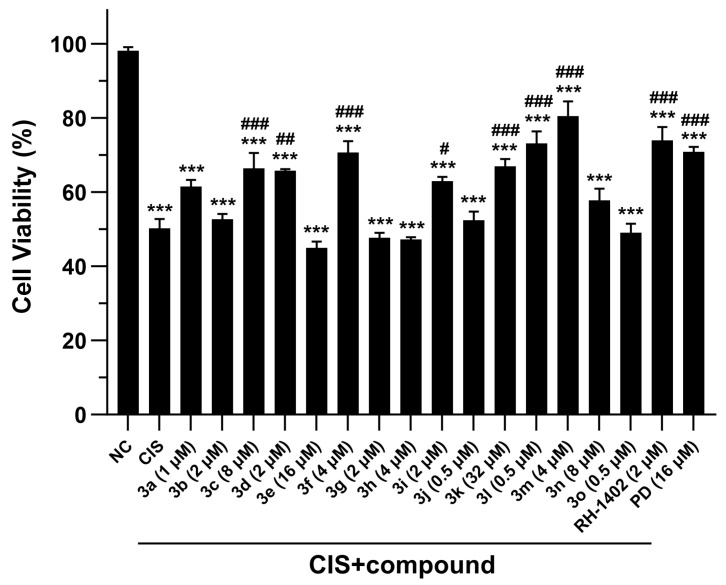
The most effective protection of target compounds on mRTEC viability under CIS treatment. *** *p* < 0.001 vs. NC; # *p* < 0.05, ## *p* < 0.01, ### *p* < 0.001 vs. CIS. NC, normal control; CIS, cisplatin; PD, PD176252.

**Figure 3:**
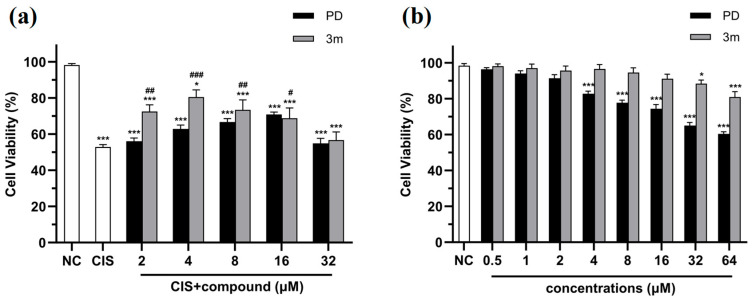
(**a**) Effects of PD176252 and **3m** on mRTEC viability under CIS treatment. (**b**) Cytotoxicity of PD176252 and **3m** at varying concentrations on mRTECs. * *p* < 0.05, *** *p* < 0.001 vs. NC; # *p* < 0.05, ## *p* < 0.01, ### *p* < 0.001 vs. CIS. NC, normal control; CIS, cisplatin; PD, PD176252.

**Figure 4:**
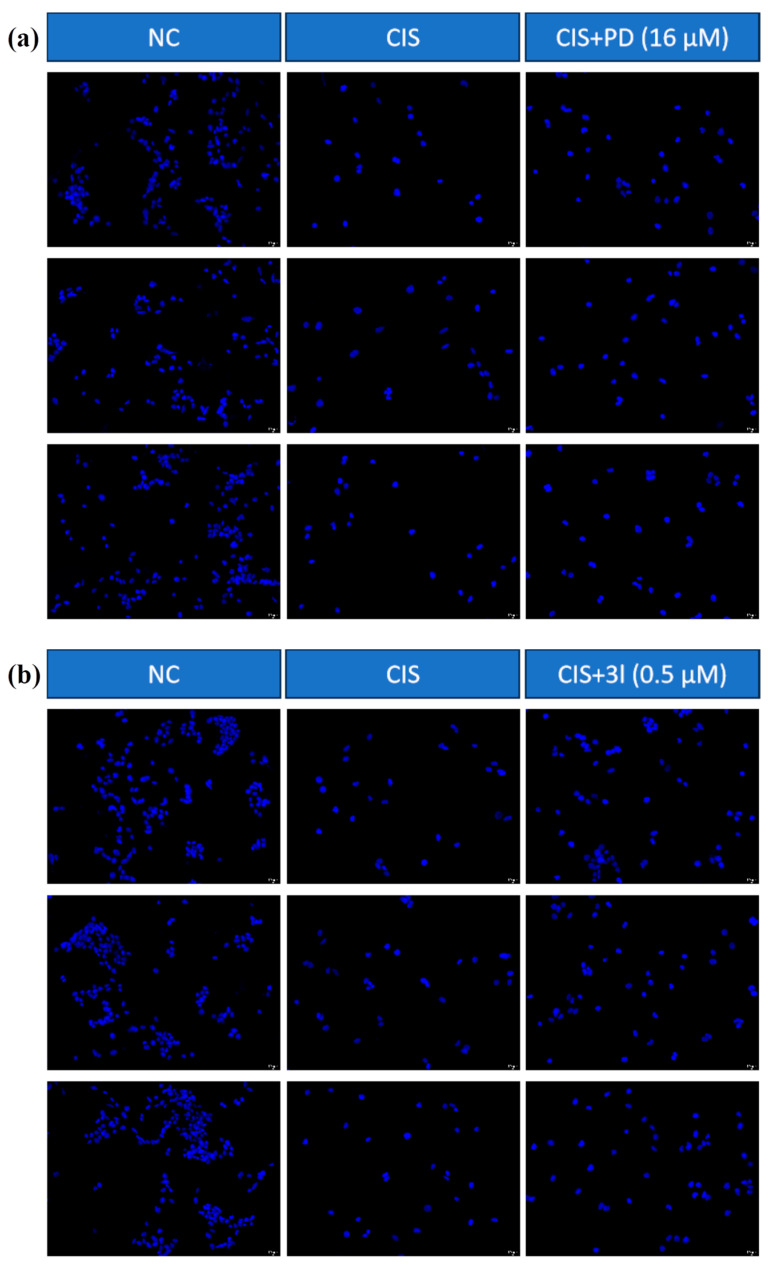

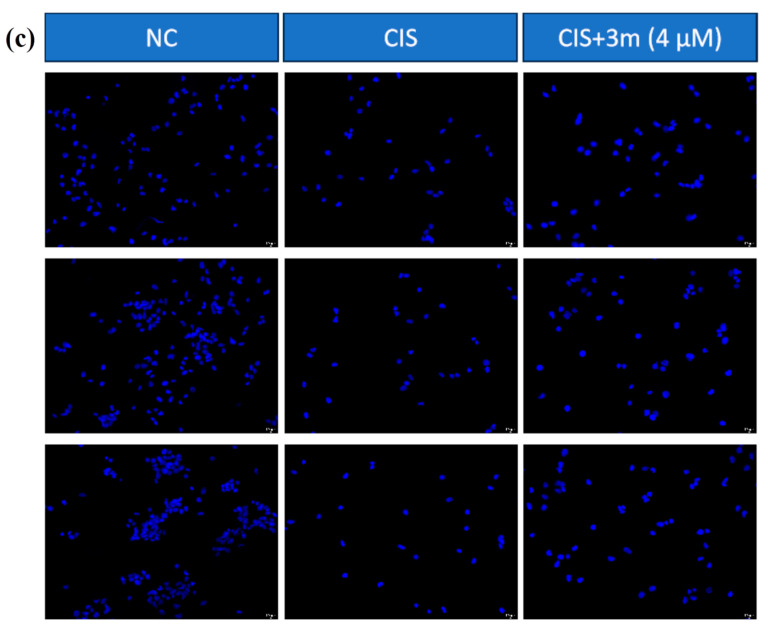
DAPI staining assay. (**a**–**c**) Protective effects of PD176252, **3l**, and **3m** on mRTECs. NC, normal control; CIS, cisplatin; PD, PD176252; magnified 50 times under microscope.

Figure 2 illustrates the compounds’ most effective protective effects against CIS-induced mRTEC death. Figure 3 depicts the protective effects of compounds PD176252 and **3m** against CIS-induced nephrotoxicity at different concentrations, alongside their cytotoxicity in normal cells. The positive control PD176252 demonstrated a maximum cell viability of 70.8 ± 1.4% at 16 µM. RH-1402 exhibited a maximum cell viability of 73.9 ± 3.7% at 2 µM. Notably, compound **3m** displayed the strongest protective effect, with a maximum cell viability reaching 80.5 ± 3.9% at 4 µM. **3m** increased cell viability by approximately 30% compared to the CIS control group. Furthermore, compared to PD176252 and RH-1402, **3m** enhanced cell viability by approximately 10%. These results suggest that **3m** is more potent than both PD176252 and RH-1402, exhibits a concentration-dependent protective effect, and shows no significant cytotoxicity. The 4′,6-diamidino-2-phenylindole (DAPI) staining assay corroborated these findings (Figure 4).

The results of the cell viability assay indicate that the protective activity of compounds **3b** and **3j** (substituted with (*R*)-configured 2-arylpropanoic acid) is lower than that of compounds **3a** and **3i** (substituted with racemic 2-arylpropanoic acid). Compound **3c** (substituted with (*S*)-configured 2-arylpropanoic acid) exhibits slightly higher protective activity than compound **3a**. Compounds with multiple substituents on the benzene ring (**3g**, **3h**, **3n**) exhibited reduced activity, while those with para-monosubstituents (**3d**, **3f**) showed enhanced activity. Para-position electron-donating groups were superior to electron-withdrawing groups, as evidenced by **3f** being more effective than **3d**. Compounds with meta-position electron-withdrawing substituents on the benzene ring (**3e**, **3g**, **3h**, **3n**) exhibited significantly reduced activity. However, substituting the benzene ring in **3a** with either benzophenone (**3l**) or naphthalene (**3m**) significantly improved cell viability. Carbazole substitution (**3o**) resulted in a notable decrease in activity. The naphthyl and methoxy groups in compound **3m** increase the electron density of the molecule through their electron-donating effects, which may facilitate π-π interactions between the molecule and biological targets, thereby enhancing the affinity between the molecule and its target. Furthermore, the presence of naphthyl and methoxy groups increases the compound’s hydrophobicity and lipophilicity, enhancing its permeability across cell membranes and thereby improving its distribution and absorption in cells. Consequently, compound **3m** demonstrates superior protective activity. In contrast, in compound **3l**, the phenyl group has weaker conjugation and lower electron density compared to the naphthyl group in **3m**, leading to a weaker binding affinity to the target. Although the phenyl ketone group also exerts electronic effects, its electron-donating capacity is less pronounced than that of methoxy. Moreover, the relatively simple molecular structure of the phenyl ketone group may limit its compatibility with the target. As a result, the activity of **3l** is lower than that of **3m**.

#### 2.2.2. Anti-Inflammatory Activity of Compounds **3m** and **3l**

Kidney injury molecule-1 (KIM-1) is a transmembrane protein primarily expressed by proximal tubular epithelial cells [35]. Following kidney injury, KIM-1 levels are significantly elevated, establishing it as a reliable biomarker for assessing the renal protective effects of various compounds [35]. Inflammatory mediators, such as tumor necrosis factor-α (TNF-α), interleukin-6 (IL-6), and monocyte chemoattractant protein-1 (MCP-1), play a critical role in the pathogenesis of CIS-induced kidney damage [36,37,38]. To evaluate whether the new compound mitigates CIS-induced nephrotoxicity by inhibiting the inflammatory response, real-time polymerase chain reaction (PCR) was employed to detect the mRNA expression levels of KIM-1 and inflammatory cytokines.

**Figure 5:**
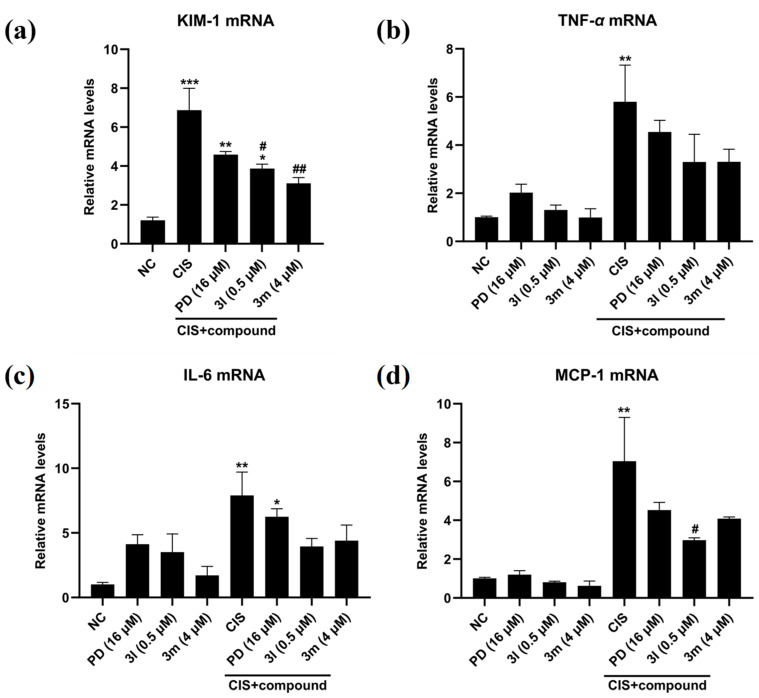
(**a**) Effects of PD176252, **3l**, and **3m** on KIM-1 in CIS-treated mRTEC. (**b**–**d**) Effects of PD176252, **3l**, and **3m** on inflammatory cytokines in CIS-treated and untreated mRTEC. * *p* < 0.05, ** *p* < 0.01, *** *p* < 0.001 vs. NC; # *p* < 0.05, ## *p* < 0.01 vs. CIS. NC, normal control; CIS, cisplatin; PD, PD176252.

The results indicated that compared to normal cells, CIS treatment alone significantly increased the expression of KIM-1 and inflammatory cytokines. Following co-treatment with CIS and target compounds, the mRNA levels of KIM-1, TNF-α, IL-6, and MCP-1 were significantly reduced. Compounds **3l** and **3m** exhibited more pronounced inhibitory effects on KIM-1, TNF-α, and IL-6 in comparison to PD176252 (Figure 5).

Our previous studies have demonstrated that PD176252 and RH-1402 lower the levels of KIM-1 and inflammatory cytokines through the inhibition of the NF-κB signaling pathway [21,27]. Real-time PCR experiments suggest that the downregulation of KIM-1 expression observed with **3l** and **3m** implies they possess a protective effect on the kidneys, alleviating CIS-induced kidney injury, mitigating damage to tubular cells, and reducing apoptosis or necrosis, thereby decreasing the expression of damage markers. The observed reduction in the levels of inflammatory factors (TNF-α, IL-6, and MCP-1) indicates that the protective effects of compounds **3l** and **3m** may be attributed to their capacity to inhibit the NF-κB signaling pathway, leading to a reduction in the transcription of inflammatory cytokine genes and the stability of their mRNA. Furthermore, their effects appear to be superior to those of PD176252.

### 2.3. SwissADME Predictions

SwissADME is a freely accessible online tool used to assess the pharmacokinetics, drug similarity, and chemical properties of small molecules [39,40,41]. The predictions provided by SwissADME version 2024, as shown in Table 1, reveal that the log *P*_o_/*w* values (partition coefficient between *n*-octanol and water) for both compounds **3l** and **3m** are below five, indicating favorable membrane permeability. Both **3l** and **3m** demonstrate superior gastrointestinal absorption and higher bioavailability scores compared to PD176252. Moreover, the AMES toxicity of compounds **3l** and **3m** is lower than that of both PD176252 and RH-1402. Furthermore, both **3l** and **3m** adhere to Lipinski’s rules. In summary, compounds **3l** and **3m** exhibit outstanding pharmacokinetic profiles and drug similarity.

### 2.4. Analysis of Molecular Docking Results

**Figure 6:**
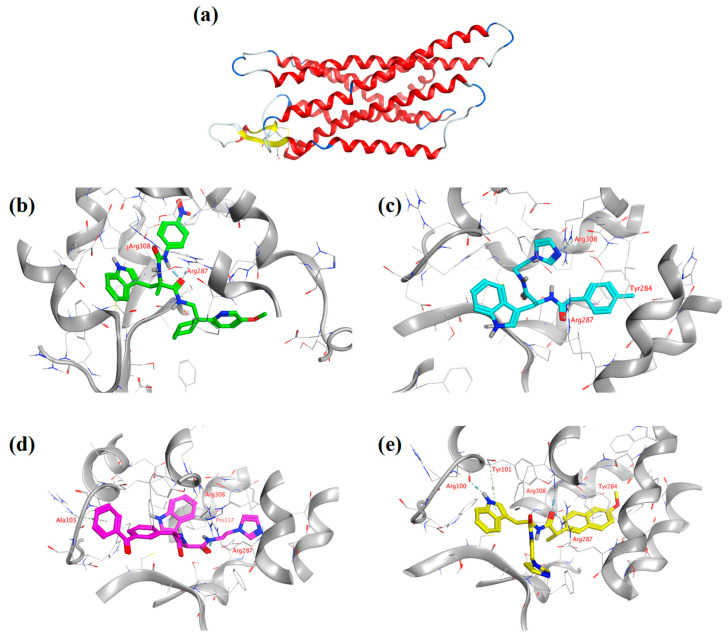
(**a**) Homology model of the GRPR protein; (**b**) molecular docking results of PD176252 (green) to the GRPR; (**c**) molecular docking results of RH-1402 (blue) to the GRPR; (**d**) molecular docking results of **3l** (purple) to the GRPR; (**e**) molecular docking results of **3m** (yellow) to the GRPR.

To investigate the interaction between the GRPR and target compounds, a homology model of the GRPR protein was constructed using Discovery Studio (DS) software version 2023 [27]. Molecular docking simulations were subsequently performed with the Molecular Operating Environment (MOE) software version 2024.06, and the results are presented in Figure 6. The docking score serves as a critical indicator of the molecular docking outcomes, representing the affinity between the ligand and the protein. A lower docking score indicates a stronger affinity. Furthermore, an increased number of hydrogen bonds typically correlates with more stable interactions between the ligand and the protein. The docking scores for PD176252, RH-1402, **3l**, and **3m** were −7.9727, −7.7658, −8.5718, and −8.2977, respectively. Compound **3m** formed three hydrogen bonds with Arg100, Arg287, and Arg308, a π-H interaction with Tyr101, and a π-π interaction with Tyr284. Compound **3l** formed one hydrogen bond with Arg287, two π-H interactions with Ala103 and Pro117, and a π-cation interaction with Arg308. In contrast, PD176252 formed only two hydrogen bonds with Arg287 and Arg308. RH-1402, similarly, formed two hydrogen bonds with Arg287 and Arg308, along with a π-π interaction with Tyr284. Therefore, both **3m** and **3l** exhibited stronger binding affinities compared to PD176252 and RH-1402.

## 3. Materials and Methods

### 3.1. Chemistry

All chemicals and reagents, including the 2-arylpropionic acid derivatives (**a**–**o**), were sourced from commercial suppliers (Aladdin, Shanghai, China) and used without further purification. Reactions were monitored by thin-layer chromatography on silica gel GF254 plates (Aladdin, Shanghai, China), and the results were visualized under ultraviolet (254 nm) light. The ^1^H and ^13^C nuclear magnetic resonance (NMR) spectra were recorded on an Agilent VNMRS600 NMR spectrometer (Agilent, Santa Clara, CA, USA), with DMSO-d_6_ as the solvent. High-resolution mass spectrometry (HR-MS) analysis was conducted using a Thermo Fisher Scientific Vanquish Q Exactive Plus mass spectrometer (Thermo Fisher Scientific, Waltham, MA, USA). Melting points were determined using a Stuart Melting Point Apparatus (MPA) (Stuart, Vernon Hills, IL, USA).

### 3.2. Procedure for Synthesizing Compounds **3a**–**o** (Scheme 1)

#### 3.2.1. Synthesis of Intermediates **1a**–**o**

A mixture of 2-arylpropanoic acid derivatives (3.0 mmol), L-tryptophan methyl ester hydrochloride (3.3 mmol), *N*,*N*-diisopropylethylamine (DIPEA, 7.0 mmol), 1-ethyl-3-(3-dimethylaminopropyl)carbodiimide (EDCI, 3.3 mmol), 1-hydroxybenzotriazole (HOBt, 0.41 mmol), and 30 mL of dry dichloromethane (DCM) was stirred at room temperature for 7–8 h. Then, the mixture was washed with water (2 × 30 mL) and 5% hydrochloric acid (1 × 30 mL). The organic layer was concentrated under reduced pressure and purified via silica gel column chromatography (DCM/ethyl acetate), resulting in intermediates **1a**–**o** with yields ranging from 80.2% to 88.6%.

#### 3.2.2. Synthesis of Intermediates **2a**–**o**

The intermediates **1a**–**o** (2.0 mmol) were subjected to hydrolysis in the presence of Na_2_CO_3_ (6.0 mmol), methanol (MeOH, 20 mL), and water (7 mL) at 55 °C for 5–6 h. After cooling to room temperature, the pH was adjusted to 1–2 using 5% hydrochloric acid, followed by filtration and washing with water to produce intermediates **2a**–**o** with yields ranging from 81.2% to 86.5%.

#### 3.2.3. Synthesis of Target Compounds **3a**–**o**

A mixture of intermediates **2a**–**o** (1.0 mmol), 2-(1*H*-imidazol-1-yl)ethan-1-amine (1.1 mmol), EDCI (1.1 mmol), HOBt (0.14 mmol), DIPEA (2.3 mmol), and DCM (10 mL) was stirred at room temperature for 24 h. The mixture was washed with water (2 × 30 mL) and concentrated under reduced pressure. The rough product was purified by recrystallization (MeOH/H_2_O) or column chromatography (DCM/MeOH), resulting in target product **3a**–**o** with yields ranging from 30.2% to 55.6%.

### 3.3. Structural Characterization of Target Compounds **3a**–**o**

**3a**: white solid, yield: 51.2%. MP: 312.3–313.5 °C; ^1^H NMR (600 MHz, DMSO-d_6_) *δ* 10.74 (d, *J* = 2.1 Hz, 1H), 8.20 (t, *J* = 5.5 Hz, 1H), 8.15 (d, *J* = 8.1 Hz, 1H), 7.56 (s, 1H), 7.51 (d, *J* = 7.8 Hz, 1H), 7.30 (d, *J* = 8.1 Hz, 1H), 7.24–7.19 (m, 2H), 7.19–7.15 (m, 3H), 7.08 (s, 1H), 7.03 (t, *J* = 7.5 Hz, 1H), 6.93 (t, *J* = 7.4 Hz, 1H), 6.91–6.86 (m, 2H), 4.41 (td, *J* = 8.3, 5.5 Hz, 1H), 4.01–3.91 (m, 2H), 3.71 (q, *J* = 7.2 Hz, 1H), 3.38 (dq, *J* = 12.4, 6.1 Hz, 1H), 3.34–3.28 (m, 1H), 2.96 (dd, *J* = 14.5, 5.4 Hz, 1H), 2.84 (dd, *J* = 14.5, 8.7 Hz, 1H), 1.30 (d, *J* = 7.0 Hz, 3H). ^13^C NMR (151 MHz, DMSO-d_6_) *δ* 173.19, 172.01, 141.91, 137.33, 136.00, 128.10, 127.40, 127.21, 126.36, 123.56, 120.83, 119.69, 118.43, 118.18, 111.24, 109.87, 53.65, 45.32, 44.47, 27.77, 18.02. HR-MS *m*/*z*: calcd. for C_25_H_28_N_5_O_2_ [M + H]^+^ 430.2238, found: 430.2230.

**3b**: white solid, yield: 41.2%. MP: 180.1–181.3 °C; ^1^H NMR (600 MHz, DMSO-d_6_) *δ* 10.78 (d, *J* = 2.4 Hz, 1H), 8.32 (s, 1H), 8.28 (d, *J* = 7.1 Hz, 1H), 7.51 (d, *J* = 7.9 Hz, 1H), 7.38 (d, *J* = 1.6 Hz, 1H), 7.30 (d, *J* = 8.1 Hz, 1H), 7.28–7.25 (m, 4H), 7.23–7.19 (m, 2H), 7.17–7.15 (m, 2H), 6.93 (t, *J* = 7.3 Hz, 1H), 6.90 (d, *J* = 2.4 Hz, 1H), 4.35 (td, *J* = 8.4, 5.4 Hz, 1H), 4.10 (q, *J* = 6.1 Hz, 2H), 3.73–3.68 (m, 1H), 3.42 (t, *J* = 6.3 Hz, 1H), 3.36 (dd, *J* = 12.1, 5.9 Hz, 1H), 2.97 (dd, *J* = 14.5, 5.2 Hz, 1H), 2.85 (dd, *J* = 14.6, 8.9 Hz, 1H), 1.29 (d, *J* = 7.0 Hz, 3H). ^13^C NMR (151 MHz, DMSO-d_6_) *δ* 173.34, 172.22, 141.86, 136.39, 136.01, 128.11, 127.44, 127.21, 126.38, 123.61, 121.08, 120.84, 118.40, 118.20, 111.29, 109.87, 53.87, 46.81, 44.46, 27.69, 17.97. HR-MS *m*/*z*: calcd. for C_25_H_28_N_5_O_2_ [M + H]^+^ 430.2238, found: 430.2236.

**3c**: white solid, yield: 42.3%. MP: 180.2–182.1 °C; ^1^H NMR (600 MHz, DMSO-d_6_) *δ* 10.76 (s, 1H), 8.26 (d, *J* = 7.2 Hz, 1H), 8.21 (s, 1H), 7.60 (d, *J* = 7.9 Hz, 1H), 7.50 (d, *J* = 7.9 Hz, 1H), 7.33 (d, *J* = 7.6 Hz, 1H), 7.29 (d, *J* = 8.1 Hz, 1H), 7.27–7.26 (m, 2H), 7.22–7.19 (m, 2H), 7.18–7.14 (m, 3H), 6.93 (t, *J* = 7.4 Hz, 1H), 6.89 (d, *J* = 2.3 Hz, 1H), 4.35 (dt, *J* = 8.4, 4.2 Hz, 1H), 4.10–4.06 (m, 2H), 3.71 (d, *J* = 7.1 Hz, 1H), 3.40 (d, *J* = 5.1 Hz, 1H), 3.34 (dd, *J* = 14.8, 5.8 Hz, 1H), 2.96 (dd, *J* = 14.5, 5.3 Hz, 1H), 2.84 (dd, *J* = 14.6, 8.9 Hz, 1H), 1.29 (d, *J* = 7.0 Hz, 3H). ^13^C NMR (151 MHz, DMSO-d_6_) *δ* 173.33, 172.22, 141.85, 136.32, 136.00, 128.10, 127.42, 127.20, 126.37, 123.60, 121.15, 120.83, 118.39, 118.19, 111.27, 109.86, 53.87, 46.90, 44.45, 27.67, 17.96. HR-MS *m*/*z*: calcd. for C_25_H_28_N_5_O_2_ [M + H]^+^ 430.2238, found: 430.2233.

**3d**: light yellow solid, yield: 30.5%. MP: 320.5–321.9 °C; ^1^H NMR (600 MHz, DMSO-d_6_) *δ* 10.70 (d, *J* = 2.5 Hz, 1H), 8.36 (d, *J* = 8.1 Hz, 1H), 8.29 (t, *J* = 5.7 Hz, 1H), 8.04–7.96 (m, 2H), 7.74 (s, 1H), 7.49 (d, *J* = 7.9 Hz, 1H), 7.35–7.29 (m, 2H), 7.25 (d, *J* = 8.1 Hz, 1H), 7.18 (s, 1H), 7.03–6.96 (m, 2H), 6.90 (ddd, *J* = 8.0, 6.9, 1.0 Hz, 1H), 6.87 (d, *J* = 2.3 Hz, 1H), 4.44 (ddd, *J* = 9.6, 8.2, 5.0 Hz, 1H), 4.07–3.98 (m, 2H), 3.86 (q, *J* = 7.1 Hz, 1H), 3.42 (dq, *J* = 12.3, 5.8 Hz, 1H), 3.39–3.34 (m, 1H), 2.97 (dd, *J* = 14.6, 4.9 Hz, 1H), 2.81 (dd, *J* = 14.6, 9.6 Hz, 1H), 1.31 (d, *J* = 7.0 Hz, 3H). ^13^C NMR (151 MHz, DMSO-d_6_) *δ* 171.98, 171.72, 149.68, 146.08, 135.97, 128.76, 128.28, 127.09, 123.56, 123.33, 123.18, 120.77, 118.40, 118.13, 111.16, 109.78, 53.56, 45.65, 44.42, 27.85, 17.75. HR-MS *m*/*z*: calcd. for C_25_H_27_N_6_O_4_ [M + H]^+^ 475.2088, found: 475.2086.

**3e**: white solid, yield: 35.5%. MP: 291.1–292.8 °C; ^1^H NMR (600 MHz, DMSO-d_6_) *δ* 10.75 (d, *J* = 2.4 Hz, 1H), 8.31 (dd, *J* = 8.1, 2.4 Hz, 1H), 8.28–8.23 (m, 1H), 7.86 (s, 1H), 7.50 (d, *J* = 7.9 Hz, 1H), 7.31–7.26 (m, 2H), 7.23 (d, *J* = 6.4 Hz, 2H), 7.21 (d, *J* = 8.2 Hz, 1H), 7.11–7.08 (m, 1H), 7.04–7.01 (m, 2H), 6.94–6.90 (m, 2H), 4.38 (td, *J* = 8.3, 5.4 Hz, 1H), 4.04–3.99 (m, 2H), 3.74 (q, *J* = 7.3 Hz, 1H), 3.43–3.33 (m, 2H), 2.97 (dd, *J* = 14.5, 5.4 Hz, 1H), 2.84 (dd, *J* = 14.5, 8.7 Hz, 1H), 1.29 (d, *J* = 7.0 Hz, 3H). ^13^C NMR (151 MHz, DMSO-d_6_) *δ* 172.67, 171.98, 144.35, 136.94, 136.00, 132.78, 129.94, 127.32, 127.11, 126.42, 125.98, 123.48, 120.83, 120.23, 118.38, 118.17, 111.28, 109.86, 53.81, 45.91, 44.16, 27.78, 17.96. HR-MS *m*/*z*: calcd. for C_25_H_27_ClN_5_O_2_ [M + H]^+^ 464.1848, found: 464.1849.

**3f**: white solid, yield: 55.2%. MP: 321.6–323.2 °C; ^1^H NMR (600 MHz, DMSO-d_6_) *δ* 10.73 (d, *J* = 2.4 Hz, 1H), 8.15 (t, *J* = 5.6 Hz, 1H), 8.04 (d, *J* = 7.9 Hz, 1H), 7.52–7.46 (m, 2H), 7.29 (dt, *J* = 8.1, 0.9 Hz, 1H), 7.06–7.02 (m, 4H), 7.02–6.99 (m, 2H), 6.92 (ddd, *J* = 7.9, 6.9, 1.0 Hz, 1H), 6.89 (d, *J* = 2.3 Hz, 1H), 6.84 (t, *J* = 1.1 Hz, 1H), 4.38 (td, *J* = 8.2, 5.6 Hz, 1H), 3.96–3.90 (m, 2H), 3.64 (q, *J* = 7.2 Hz, 2H), 3.33–3.26 (m, 1H), 2.94 (dd, *J* = 14.5, 5.6 Hz, 1H), 2.82 (dd, *J* = 14.6, 8.5 Hz, 1H), 2.24 (s, 3H), 1.26 (d, *J* = 7.1 Hz, 3H). ^13^C NMR (151 MHz, DMSO-d_6_) *δ* 173.35, 171.99, 138.90, 137.37, 136.01, 135.28, 128.67, 128.30, 127.21, 127.07, 123.56, 120.83, 119.59, 118.44, 118.18, 111.23, 109.88, 53.63, 45.21, 44.09, 39.98, 27.77, 20.68, 18.08. HR-MS *m*/*z*: calcd. for C_26_H_30_N_5_O_2_ [M + H]^+^ 444.2394, found: 444.2391.

**3g**: light yellow solid, yield: 30.2%. MP: 291.3–293.1 °C; ^1^H NMR (600 MHz, DMSO-d_6_) *δ* 10.69 (d, *J* = 2.5 Hz, 1H), 8.35 (dd, *J* = 8.0, 1.5 Hz, 1H), 8.28 (t, *J* = 5.7 Hz, 1H), 8.00 (s, 1H), 7.83 (d, *J* = 1.8 Hz, 1H), 7.47 (d, *J* = 7.9 Hz, 1H), 7.33 (dd, *J* = 7.9, 2.0 Hz, 1H), 7.30–7.23 (m, 3H), 7.11 (s, 1H), 7.02–6.98 (m, 1H), 6.91–6.86 (m, 2H), 4.39 (td, *J* = 8.4, 5.3 Hz, 1H), 4.08–4.02 (m, 2H), 3.81 (q, *J* = 7.0 Hz, 2H), 3.42–3.38 (m, 1H), 2.97 (dd, *J* = 14.5, 5.3 Hz, 1H), 2.82 (dd, *J* = 14.5, 8.9 Hz, 1H), 2.47 (s, 3H), 1.30 (d, *J* = 7.0 Hz, 3H). ^13^C NMR (151 MHz, DMSO-d_6_) *δ* 172.46, 171.96, 148.49, 141.42, 136.76, 135.96, 132.58, 132.25, 130.93, 127.10, 125.50, 123.46, 122.84, 120.76, 120.46, 118.33, 118.10, 111.21, 109.80, 53.78, 46.17, 43.65, 39.57, 27.79, 19.38, 17.99. HR-MS *m*/*z*: calcd. for C_26_H_29_N_6_O_4_ [M + H]^+^ 489.2245, found: 489.2238.

**3h**: yellow solid, yield: 50.0%. MP: 310.3–312.1 °C; ^1^H NMR (600 MHz, DMSO-d_6_) *δ* 10.60 (d, *J* = 2.5 Hz, 1H), 8.44 (d, *J* = 8.2 Hz, 1H), 8.30 (t, *J* = 5.6 Hz, 1H), 8.00 (s, 2H), 7.52 (s, 1H), 7.47–7.42 (m, 1H), 7.21 (d, *J* = 8.1 Hz, 1H), 7.07 (s, 1H), 6.98–6.95 (m, 1H), 6.89–6.82 (m, 3H), 4.46 (td, *J* = 8.5, 5.1 Hz, 1H), 3.98 (tt, *J* = 12.3, 6.1 Hz, 2H), 3.94–3.84 (m, 2H), 3.36–3.30 (m, 1H), 2.96 (dd, *J* = 14.5, 5.3 Hz, 1H), 2.80 (dd, *J* = 14.5, 9.0 Hz, 1H), 2.43 (s, 3H), 1.32 (d, *J* = 7.0 Hz, 3H). ^13^C NMR (151 MHz, DMSO-d_6_) *δ* 171.77, 171.52, 150.62, 142.73, 137.42, 135.92, 128.35, 127.11, 126.66, 124.56, 123.44, 120.74, 119.62, 118.32, 118.05, 111.18, 109.75, 53.69, 45.24, 43.62, 39.99, 28.02, 18.13, 14.41. HR-MS *m*/*z*: calcd. for C_26_H_28_N_7_O_6_ [M + H]^+^ 534.2096, found: 534.2094.

**3i**: white solid, yield: 50.5%. MP: 221.5–222.6 °C; ^1^H NMR (600 MHz, DMSO-d_6_) *δ* 10.74 (d, *J* = 2.5 Hz, 1H), 8.16 (t, *J* = 5.5 Hz, 1H), 8.06 (d, *J* = 8.0 Hz, 1H), 7.52–7.47 (m, 2H), 7.28 (d, *J* = 8.1 Hz, 1H), 7.06 (d, *J* = 8.2 Hz, 3H), 7.03–7.00 (m, 1H), 6.99 (s, 1H), 6.98 (s, 1H), 6.95–6.90 (m, 2H), 6.84 (d, *J* = 1.1 Hz, 1H), 4.40 (d, *J* = 5.7 Hz, 1H), 3.96–3.90 (m, 2H), 3.66 (d, *J* = 7.1 Hz, 1H), 3.36–3.32 (m, 1H), 3.30 (d, *J* = 5.7 Hz, 1H), 2.95 (dd, *J* = 14.5, 5.6 Hz, 1H), 2.83 (dd, *J* = 14.5, 8.5 Hz, 1H), 2.37 (d, *J* = 7.2 Hz, 2H), 1.84–1.69 (m, 1H), 1.27 (d, *J* = 7.1 Hz, 3H), 0.83 (d, *J* = 6.6 Hz, 6H). ^13^C NMR (151 MHz, DMSO-d_6_) *δ* 173.35, 171.98, 139.11, 139.03, 137.36, 136.03, 128.65, 128.29, 127.20, 126.92, 123.63, 120.80, 119.58, 118.45, 118.16, 111.23, 109.86, 53.63, 45.21, 44.31, 44.09, 39.98, 29.63, 27.80, 22.25, 17.98. HR-MS *m*/*z*: calcd. for C_29_H_36_N_5_O_2_ [M + H]^+^ 486.2864, found: 486.2858.

**3j**: white solid, yield: 43.3%. MP: 170.6–172.3 °C; ^1^H NMR (600 MHz, DMSO-d_6_) *δ* 10.74 (d, *J* = 2.4 Hz, 1H), 8.15 (d, *J* = 5.2 Hz, 1H), 8.06 (d, *J* = 8.0 Hz, 1H), 7.52–7.48 (m, 2H), 7.29 (d, *J* = 8.1 Hz, 1H), 7.07 (s, 1H), 7.05 (d, *J* = 2.5 Hz, 2H), 6.99 (s, 1H), 6.98 (d, *J* = 4.2 Hz, 2H), 6.94–6.92 (m, 2H), 6.84 (d, *J* = 1.1 Hz, 1H), 4.40 (td, *J* = 8.2, 5.7 Hz, 1H), 3.97–3.91 (m, 2H), 3.67–3.63 (m, 1H), 3.30 (dd, *J* = 6.0, 3.8 Hz, 1H), 3.27–3.22 (m, 1H), 2.95 (dd, *J* = 14.6, 5.6 Hz, 1H), 2.84 (dd, *J* = 14.5, 8.5 Hz, 1H), 2.37 (d, *J* = 7.1 Hz, 2H), 1.79–1.76 (m, 1H), 1.28 (d, *J* = 7.1 Hz, 3H), 0.83 (d, *J* = 3.0 Hz, 6H). ^13^C NMR (151 MHz, DMSO-d_6_) *δ* 173.35, 171.98, 139.11, 139.03, 137.35, 136.02, 128.65, 128.28, 127.20, 126.92, 123.62, 120.80, 119.57, 118.44, 118.16, 111.22, 109.86, 53.63, 45.21, 44.27, 44.09, 39.97, 29.64, 27.79, 22.25, 17.98. HR-MS *m*/*z*: calcd. for C_29_H_36_N_5_O_2_ [M + H]^+^ 486.2864; found: 486.2858.

**3k**: white solid, yield: 50.6%. MP: 161.2–162.8 °C; ^1^H NMR (600 MHz, DMSO-d_6_) *δ* 10.74 (s, 1H), 8.17 (t, *J* = 5.8 Hz, 1H), 8.07 (dd, *J* = 8.1, 3.0 Hz, 1H), 7.50 (d, *J* = 8.0 Hz, 2H), 7.31–7.27 (m, 1H), 7.19–7.15 (m, 1H), 7.07 (d, *J* = 8.0 Hz, 4H), 7.03–7.01 (m, 2H), 6.92 (t, *J* = 2.6 Hz, 1H), 6.85 (s, 1H), 4.45–4.36 (m, 1H), 3.95 (dq, *J* = 9.3, 6.4, 5.8 Hz, 2H), 3.88 (t, *J* = 6.1 Hz, 1H), 3.68–3.64 (m, 1H), 2.94 (q, *J* = 3.5, 3.0 Hz, 1H), 2.93–2.91 (m, 1H), 2.84 (dd, *J* = 14.5, 8.6 Hz, 1H), 2.43–2.30 (m, 3H), 2.26–2.19 (m, 1H), 2.07 (ddd, *J* = 18.8, 10.4, 8.6 Hz, 1H), 1.85 (tdt, *J* = 12.1, 6.3, 3.2 Hz, 2H), 1.70–1.63 (m, 1H), 1.44 (dddd, *J* = 12.4, 10.7, 6.8, 3.9 Hz, 1H), 1.28 (d, *J* = 7.0 Hz, 3H). ^13^C NMR (151 MHz, DMSO-d_6_) *δ* 219.31, 173.29, 172.01, 139.45, 137.38, 136.03, 128.49, 128.43, 128.30, 127.32, 127.13, 123.58, 120.81, 119.60, 118.45, 118.17, 111.29, 110.13, 53.61, 50.05, 45.22, 44.09, 39.98, 37.62, 34.57, 28.73, 20.06, 17.94. HR-MS *m*/*z*: calcd. for C_31_H_36_N_5_O_3_ [M + H]^+^ 526.2813, found: 526.2811.

**3l**: white solid, yield: 33.8%. MP: 180.0–181.3 °C; ^1^H NMR (600 MHz, DMSO-d_6_) *δ* 10.67 (d, *J* = 2.4 Hz, 1H), 8.29 (d, *J* = 8.0 Hz, 1H), 8.20 (t, *J* = 5.6 Hz, 1H), 7.69–7.65 (m, 4H), 7.57 (dt, *J* = 7.6, 1.6 Hz, 1H), 7.53–7.46 (m, 5H), 7.42 (t, *J* = 7.6 Hz, 1H), 7.26 (d, *J* = 8.0 Hz, 1H), 7.08–7.03 (m, 1H), 7.00 (ddd, *J* = 8.1, 6.9, 1.2 Hz, 1H), 6.92–6.88 (m, 1H), 6.85 (d, *J* = 2.4 Hz, 2H), 4.42 (td, *J* = 8.2, 5.7 Hz, 1H), 4.00–3.88 (m, 2H), 3.83 (d, *J* = 7.1 Hz, 1H), 3.35–3.27 (m, 2H), 2.96 (dd, *J* = 14.5, 5.6 Hz, 1H), 2.83 (dd, *J* = 14.6, 8.4 Hz, 1H), 1.33 (d, *J* = 7.0 Hz, 3H). ^13^C NMR (151 MHz, DMSO-d_6_) *δ* 195.81, 172.80, 171.86, 142.49, 137.09, 136.78, 135.97, 132.64, 131.76, 129.66, 128.61, 128.54, 128.42, 128.07, 127.18, 123.57, 123.38, 120.82, 118.57, 118.39, 118.17, 111.25, 110.10, 109.90, 53.69, 45.21, 44.33, 39.97, 27.87, 18.28. HR-MS *m*/*z*: calcd. for C_32_H_32_N_5_O_3_ [M + H]^+^ 534.2500, found: 534.2493.

**3m**: white solid, yield: 50.8%. MP: 191.6–192.5 °C; ^1^H NMR (600 MHz, DMSO-d_6_) *δ* 10.84 (d, *J* = 2.4 Hz, 1H), 8.24 (d, *J* = 8.3 Hz, 1H), 8.13 (s, 1H), 7.72 (dd, *J* = 16.4, 8.8 Hz, 2H), 7.69–7.66 (m, 1H), 7.62 (dd, *J* = 7.9, 1.1 Hz, 1H), 7.44 (d, *J* = 1.1 Hz, 1H), 7.41 (dd, *J* = 8.5, 1.8 Hz, 1H), 7.36–7.31 (m, 1H), 7.25 (d, *J* = 2.5 Hz, 1H), 7.12 (dd, *J* = 8.8, 2.5 Hz, 2H), 7.07 (d, *J* = 1.2 Hz, 1H), 6.99 (t, *J* = 1.0 Hz, 1H), 6.94 (t, *J* = 1.2 Hz, 1H), 6.74 (t, *J* = 1.1 Hz, 1H), 4.50 (td, *J* = 8.4, 5.7 Hz, 1H), 3.87–3.79 (m, 6H), 3.28 (dq, *J* = 12.6, 6.0 Hz, 1H), 3.22 (dq, *J* = 13.6, 5.6 Hz, 1H), 3.03 (dd, *J* = 14.4, 5.6 Hz, 1H), 2.92 (dd, *J* = 14.5, 8.6 Hz, 1H), 1.27 (d, *J* = 7.0 Hz, 3H). ^13^C NMR (151 MHz, DMSO-d_6_) *δ* 173.23, 171.84, 156.97, 137.30, 137.28, 136.08, 133.11, 129.12, 128.36, 128.26, 127.41, 126.69, 126.51, 125.40, 123.59, 120.90, 119.51, 118.59, 118.54, 118.23, 111.32, 110.18, 105.67, 55.15, 53.55, 45.13, 44.59, 39.94, 28.14, 18.74. HR-MS *m*/*z*: calcd. for C_30_H_32_N_5_O_3_ [M + H]^+^ 510.2500, found: 510.2495.

**3n**: white solid, yield: 55.2%. MP: 250.1–252.0 °C; ^1^H NMR (600 MHz, DMSO-d_6_) *δ* 10.85 (d, *J* = 2.5 Hz, 1H), 8.33 (d, *J* = 8.2 Hz, 1H), 8.22 (t, *J* = 5.7 Hz, 1H), 7.63 (d, *J* = 7.9 Hz, 1H), 7.53–7.50 (m, 2H), 7.47–7.46 (m, 2H), 7.44 (d, *J* = 3.9 Hz, 1H), 7.42 (d, *J* = 8.2 Hz, 1H), 7.39 (d, *J* = 7.0 Hz, 1H), 7.34 (d, *J* = 8.0 Hz, 1H), 7.24–7.17 (m, 2H), 7.12 (d, *J* = 2.4 Hz, 1H), 7.08–7.05 (m, 1H), 7.00 (d, *J* = 7.5 Hz, 1H), 6.98 (d, *J* = 2.7 Hz, 1H), 6.79 (s, 1H), 4.55–4.47 (m, 1H), 3.90 (td, *J* = 5.9, 2.7 Hz, 2H), 3.78 (t, *J* = 7.2 Hz, 1H), 3.35–3.32 (m, 1H), 3.28 (d, *J* = 5.6 Hz, 1H), 3.04 (dd, *J* = 14.4, 5.7 Hz, 1H), 2.93 (dd, *J* = 14.5, 8.6 Hz, 1H), 1.22 (d, *J* = 7.0 Hz, 3H). ^13^C NMR (151 MHz, DMSO-d_6_) *δ* 172.56, 171.77, 158.78 (d, *J* = 245.3 Hz), 144.09 (d, *J* = 7.7 Hz), 137.29, 136.07, 135.05, 130.37 (d, *J* = 3.6 Hz), 128.72 (d, *J* = 2.8 Hz), 128.61, 128.26, 127.72, 127.38, 123.96 (d, *J* = 2.9 Hz), 123.58, 120.89, 119.51, 118.57, 118.22, 115.03, 114.88, 111.31, 110.10, 53.58, 45.17, 44.08, 28.16, 18.57. HR-MS *m*/*z*: calcd. for C_31_H_31_FN_5_O_2_ [M + H]^+^ 524.2456, found: 524.2449.

**3o**: white solid, yield: 55.6%. MP: 191.5–193.6 °C; ^1^H NMR (600 MHz, DMSO-d_6_) *δ* 11.41 (s, 1H), 10.78 (s, 1H), 8.28–8.22 (m, 2H), 8.15 (dd, *J* = 5.3, 2.1 Hz, 1H), 8.02–7.95 (m, 2H), 7.48 (dd, *J* = 8.4, 6.6 Hz, 2H), 7.42 (d, *J* = 1.4 Hz, 1H), 7.36 (dd, *J* = 8.6, 2.2 Hz, 1H), 7.27 (d, *J* = 8.1 Hz, 1H), 7.23 (s, 1H), 7.10 (s, 1H), 7.05 (dd, *J* = 8.1, 1.5 Hz, 1H), 7.01–6.97 (m, 1H), 6.95 (d, *J* = 2.4 Hz, 1H), 6.89 (t, *J* = 7.5 Hz, 1H), 4.40 (td, *J* = 7.9, 5.8 Hz, 1H), 4.08–3.97 (m, 2H), 3.91 (d, *J* = 7.0 Hz, 1H), 3.43–3.33 (m, 2H), 2.97 (dd, *J* = 14.5, 5.8 Hz, 1H), 2.87 (dd, *J* = 14.5, 8.1 Hz, 1H), 1.40 (d, *J* = 7.0 Hz, 3H). ^13^C NMR (151 MHz, DMSO-d_6_) *δ* 173.47, 172.06, 140.55, 140.51, 138.34, 135.96, 127.18, 125.36, 124.92, 123.70, 123.49, 122.73, 120.81, 120.52, 120.27, 120.14, 119.57, 118.90, 118.38, 118.17, 112.34, 111.25, 109.87, 109.64, 53.91, 46.22, 45.02, 39.60, 27.73, 18.63. HR-MS *m*/*z*: calcd. for C_31_H_30_ClN_6_O_2_ [M + H]^+^ 553.2113, found: 553.2109.

### 3.4. Cells

The mouse renal tubular epithelial cells (Thermo Fisher Scientific, Waltham, MA, USA) were cultured in F12 medium (Hyclone, Logan, UT, USA) supplemented with 10% fetal bovine serum (Hyclone, Logan, UT, USA) at 37 °C and 5% CO_2_.

### 3.5. Cell Viability Assay

Cell viability was determined using the CCK-8 assay. Mouse renal tubular epithelial cells were cultured in a 96-well plate (Thermo Fisher Scientific, Waltham, MA, USA) for 24 h. The normal control group (NC) was administered saline, and the model group (CIS) was treated with 20 µM of cisplatin. The experimental group (CIS + compound) was first treated with the target compound at different concentrations (0.5 µM–64 µM) for 12 h, followed by treatment with 20 µM cisplatin for 24 h. The cell toxicity assay involved treating the cells with the compound alone for 24 h. Then, 10 µL of CCK-8 solution (MedChemExpress, Monmouth Junction, NJ, USA) was added, and the cells were incubated for 2 h. Finally, the absorbance was measured at 450 nm using an Elx800 microplate reader (Biotek, Winooski, VT, USA).

### 3.6. DAPI Staining Assay

After the cell viability assay, the cells were fixed with 3.7% formaldehyde for 10 min, then stained with DAPI (Merck, Darmstadt, Germany) for 5 min. The excess dye was washed off with PBS buffer and the cells were observed under a fluorescence microscope (Olympus, Tokyo, Japan).

### 3.7. RNA Extraction and Real-Time PCR Assay

Following the literature [6], total RNA was extracted from mouse kidney tissue using Trizol reagent (Thermo Fisher Scientific, Waltham, MA, USA). The RNA concentration was measured using a Drop 2000 spectrophotometer (Thermo Fisher Scientific, Waltham, MA, USA). RNA was reverse-transcribed into cRNA. Detection was performed using a real-time PCR thermocycler (Bio-Rad, Hercules, CA, USA). Primer sequences are listed in Table A1 (Appendix A).

### 3.8. Experimental Methods in Molecular Docking

According to previous studies, the GRPR homology model was constructed using DS software version 2023 [27]. The chemical structure of the target compound was drawn using ChemDraw software version 2021 and subsequently imported into MOE software version 2024.06. The imported compound was then prepared by adding polar hydrogens, protonation, and energy minimization to achieve a stable conformation. The active site of GRPR was identified using the Site Finder module in MOE. Following this, a standard molecular docking procedure was performed.

### 3.9. Experimental Data Processing

The experimental data were processed using Excel software version 2021 and plotted using GraphPad Prism software version 10. The data were expressed as mean ± SD. Statistical analysis was conducted using *t*-tests or a one-way ANOVA.

## 4. Conclusions

Cisplatin (CIS) is a potent anticancer agent that is widely utilized in clinical practice. However, its nephrotoxicity remains a significant challenge, presenting long-term health risks to patients. Currently, targeted therapies aimed at mitigating this issue are limited. PD176252 and its derivative RH-1402 have demonstrated significant protective effects against CIS-induced nephrotoxicity. Building upon the structure of RH-1402, we designed and synthesized 15 novel 2-arylpropanoic acid-L-tryptophan derivatives. The chemical structures of these compounds were confirmed through MP, ^1^H NMR, ^13^C NMR, and HR-MS analysis. Cell viability and inflammatory cytokine assays were employed to assess the protective potential of these derivatives. Among them, compound **3m** exhibited the most prominent protective effect. Following CIS treatment, compound **3m** increased mRTEC viability from approximately 50% to over 80%, outperforming PD176252 and RH-1402 in terms of efficacy. SwissADME analysis further confirmed that compound **3m** possesses favorable drug-like properties, including excellent gastrointestinal absorption and low toxicity. The molecular docking results indicated that compound **3m** demonstrates a high binding affinity to the GRPR. These findings underscore **3m** as a promising drug candidate for further investigation and development.

## 5. Patents

Risheng Yao; Ming Yuan; et al. A 2-Arylpropanoic Acid-L-Tryptophan Compound, Its Preparation Method, and Uses. 2024109742068, 26 November 2024.

## Figures and Tables

**Table 1:** SwissADME prediction for the selected compounds.

Entry	GI Absorption ^a^	BBB Permeant ^b^	Bioavailability Score ^c^	AMES Toxicity ^d^	log *P*_o_/*w* ^e^	Lipinski Rules ^f^
**PD176252**	Low	No	0.17	0.918	5.43	No
**RH-1402**	High	No	0.55	0.859	2.76	Yes
**3l**	High	No	0.55	0.393	4.24	Yes
**3m**	High	No	0.55	0.800	4.17	Yes

^a^ Gastrointestinal absorption (GI absorption); ^b^ blood–brain barrier (BBB permeant). ^c^ The date was calculated using SwissADME (Bioavailability Score). ^d^ The date was calculated using ADMETlab 2.0 (AMES Toxicity). ^e^ The partition coefficient was between *n*-octanol and water (log *P*_o_/*w*), ^f^ MW ≤ 500; MLOGP ≤ 4.15; N or O ≤ 10; NH or OH ≤ 5 (Lipinski Rules).

## Data Availability

The original contributions presented in this study are included in the article/Supplementary Materials. Further inquiries can be directed to the corresponding author(s).

## Supplementary Materials

The following supporting information can be downloaded at: https://www.mdpi.com/article/10.3390/molecules30112400/s1, Figure S1: ^1^H NMR, ^13^C NMR and HR-MS spectra of target compounds (**3a**–**o**).

## Abbreviations

The following abbreviations are used in this manuscript:

GRPR	Gastrin-Releasing Peptide Receptor
mRTEC	Mouse Renal Tubular Epithelial Cell
KIM-1	Kidney Injury Molecule-1
CIS	Cisplatin
ROS	Reactive Oxygen Species
ATP	Adenosine Triphosphate
AKI	Acute Kidney Injury
GRP	Gastrin-Releasing Peptide
NSAIDs	Non-Steroidal Anti-Inflammatory Drugs
DAPI	4′,6-diamidino-2-phenylindole
EDCI	1-Ethyl-3-(3-dimethylaminopropyl)carbodiimide
HOBt	1-Hydroxybenzotriazole
TNF-*α*	Tumor Necrosis Factor-*α*
IL-6	Interleukin-6
MCP-1	Monocyte Chemoattractant Protein-1
PCR	Polymerase Chain Reaction
NMR	Nuclear Magnetic Resonance
HR-MS	High-Resolution Mass Spectrometry
MP	Melting Point
MPA	Melting Point Apparatus
DIPEA	*N*,*N*-Diisopropylethylamine
DCM	Dichloromethane
MeOH	Methanol
DS	Discovery Studio
MOE	Molecular Operating Environment

## Appendix A

**Table A1:** Primers used in real-time PCR assay.

Mouse Gene	Forward (5′-3′)	Reverse (5′-3′)
KIM-1	CAGGGAAGCCGCAGAAAA	GAGACACGGAAGGCAACCAC
TNF-*α*	CATCTTCTCAAAATTCGAGTGACAA	TGGGAGTAGACAAGGTACAACCC
IL-6	GAGGATACCACTCCCAACAGACC	AAGTGCATCATCGTTGTTCATACA
MCP-1	CTTCTGGGCCTGCTGTTCA	CCAGCCTACTCATTGGGATCA
