# Supplementary material for: Design, Synthesis, and Evaluation of New 2-Arylpropanoic Acid-l-Tryptophan Derivatives for Mitigating Cisplatin-Induced Nephrotoxicity
Source: Molecules. 2025 May 30;30(11):2400. doi: 10.3390/molecules30112400 (PMC12156107; doi:10.3390/molecules30112400)
Supplement: Supplementary file 1 [file molecules-30-02400-s001.zip › molecules-3625598-supplementary.pdf]

Design, Synthesis, and Evaluation of New 2-Arylpropanoic Acid-L-Tryptophan Derivatives for Mitigating Cisplatin-Induced Nephrotoxicity

Ming Yuan [1] , Huai Wang [1,*] , Mingjun Yu [1,2] , Sen Yao [1] and Risheng Yao [1,*]

[1] School of Food and Biological Engineering, Hefei University of Technology, Hefei 230601, China

psv1221@163.com (Ming Yuan); yuymj127@126.com (Mingjun Yu); yaosentc@163.com (S.Y.)

[2] School of Traditional Chinese Medicine, Bozhou University, Bozhou 236800, China

* Correspondence: whuai76@hfut.edu.cn (H.W.); rishengyao@163.com (R.Y.)

Figure S1 ^{1}H NMR, ^{13}C NMR and HR-MS spectra of target compounds (**3a–o**).

(2S)-N-(2-(1H-imidazol-1-yl)ethyl)-3-(1H-indol-3-yl)-2-(2-phenylpropanamido)propanamide (**3a**)

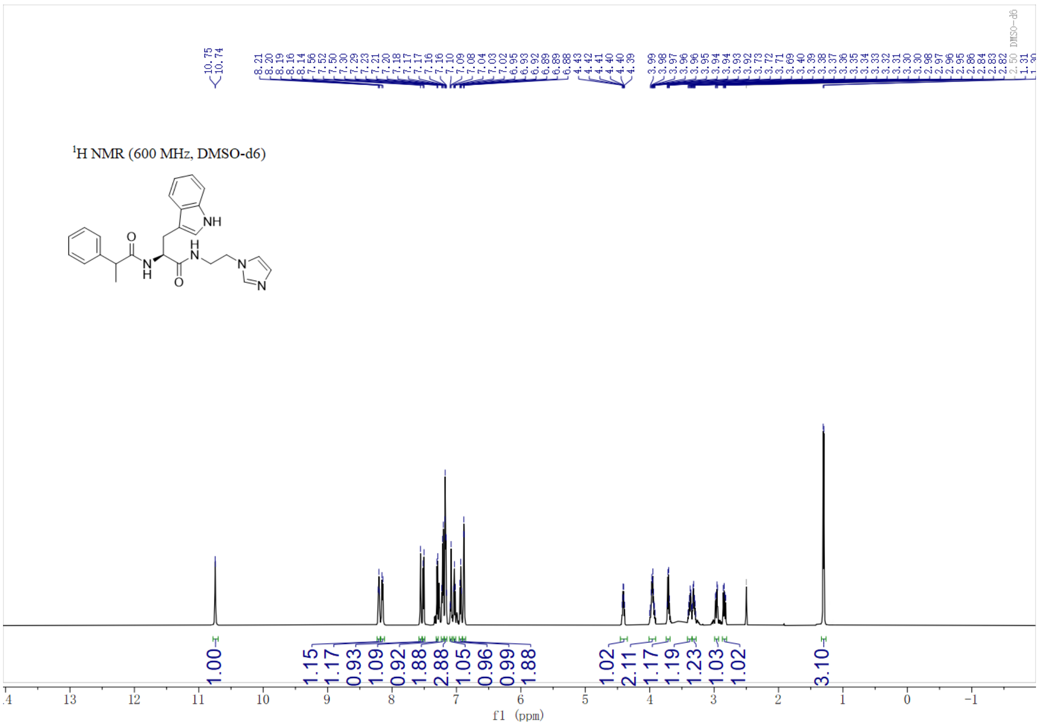

^{1}H NMR Spectrum of compound **3a**

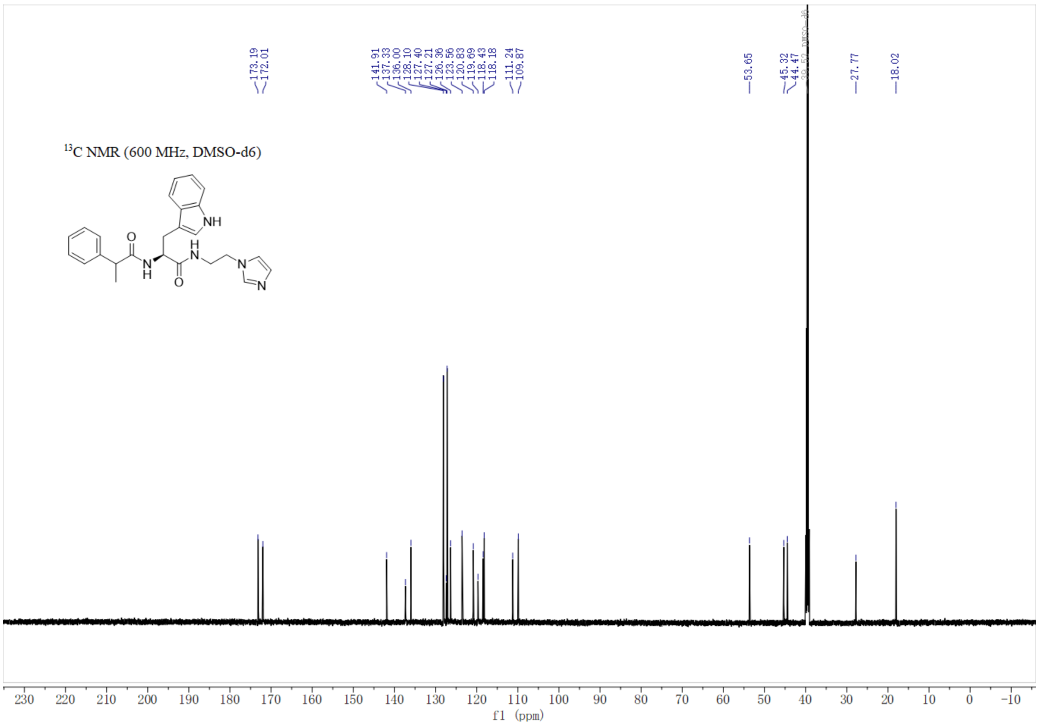

^{13}C NMR Spectrum of compound **3a**

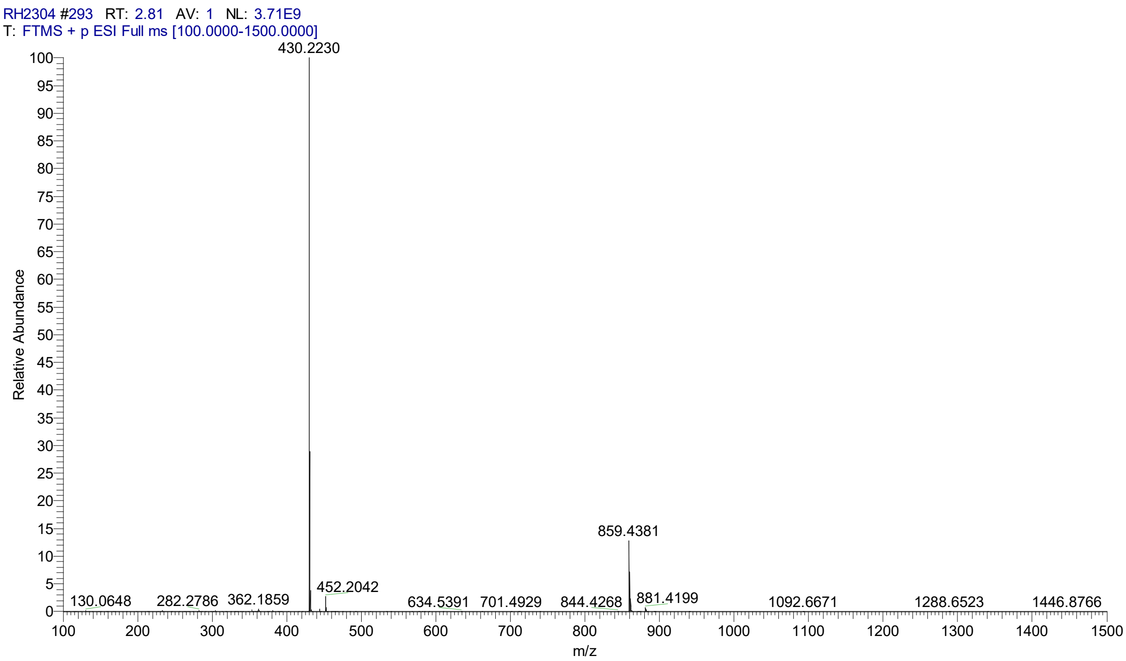

HR-MS Spectrum of compound **3a**

(S)-N-(2-(1H-imidazol-1-yl)ethyl)-3-(1H-indol-3-yl)-2-((R)-2-phenylpropanamido)propanamide (**3b**)

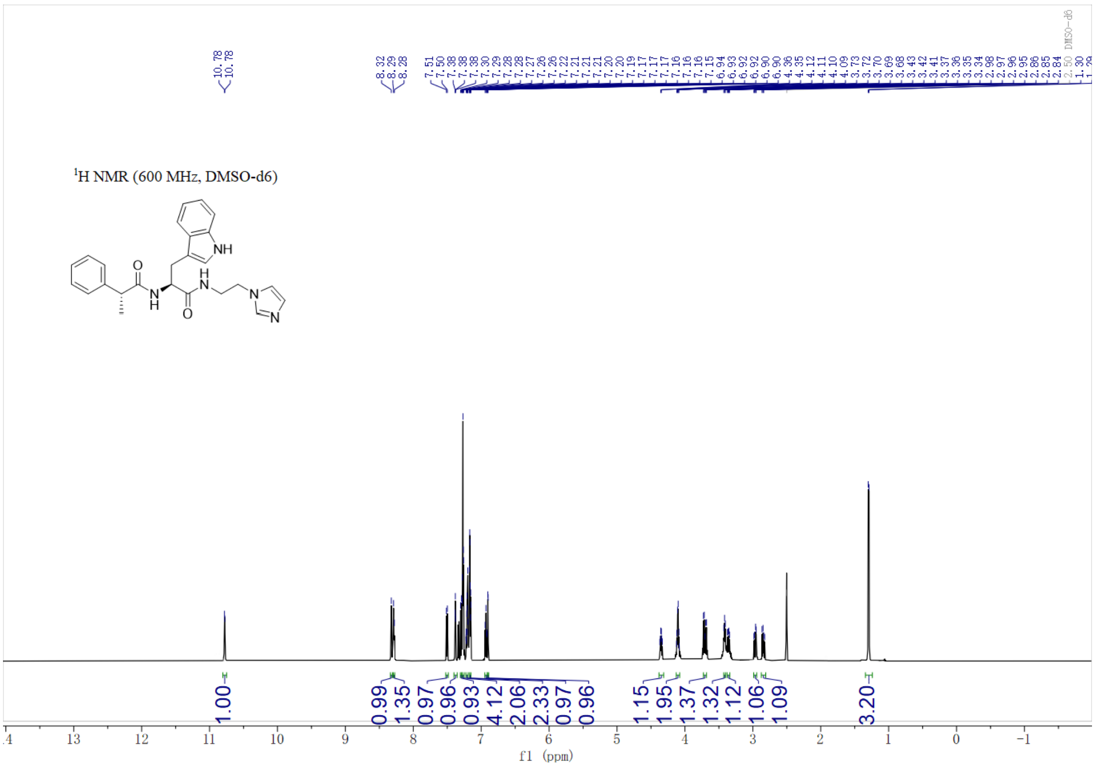

^{1}H NMR Spectrum of compound **3b**

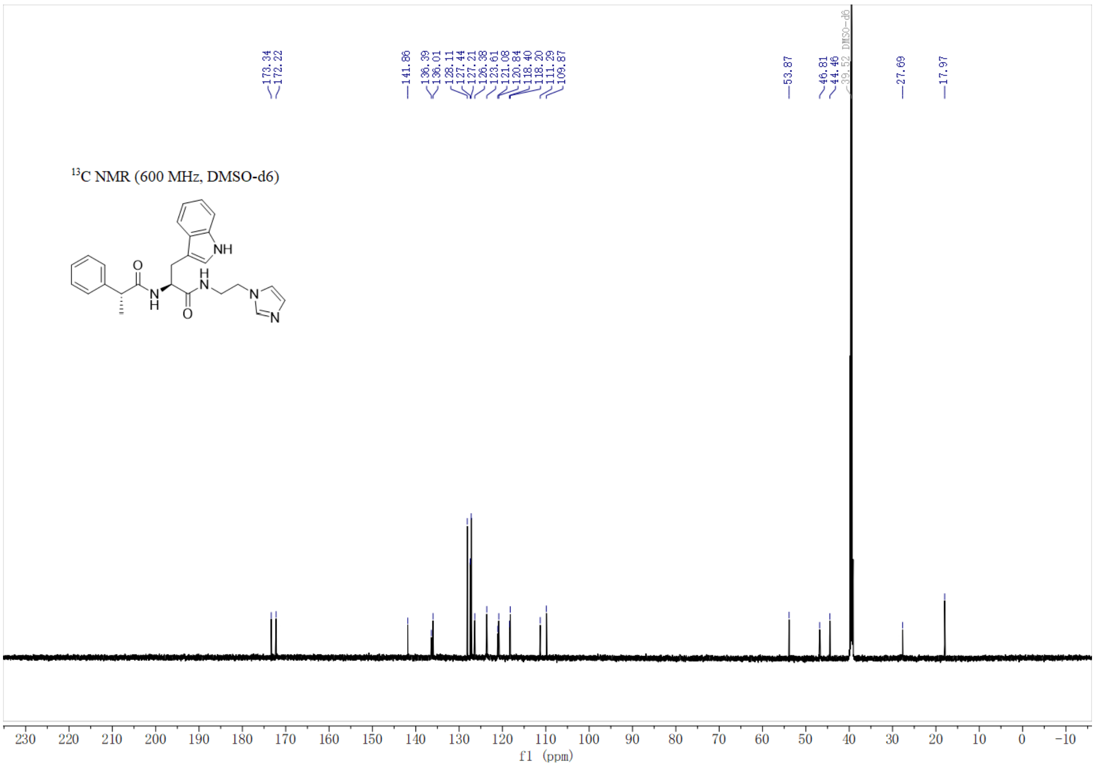

^{13}C NMR Spectrum of compound **3b**

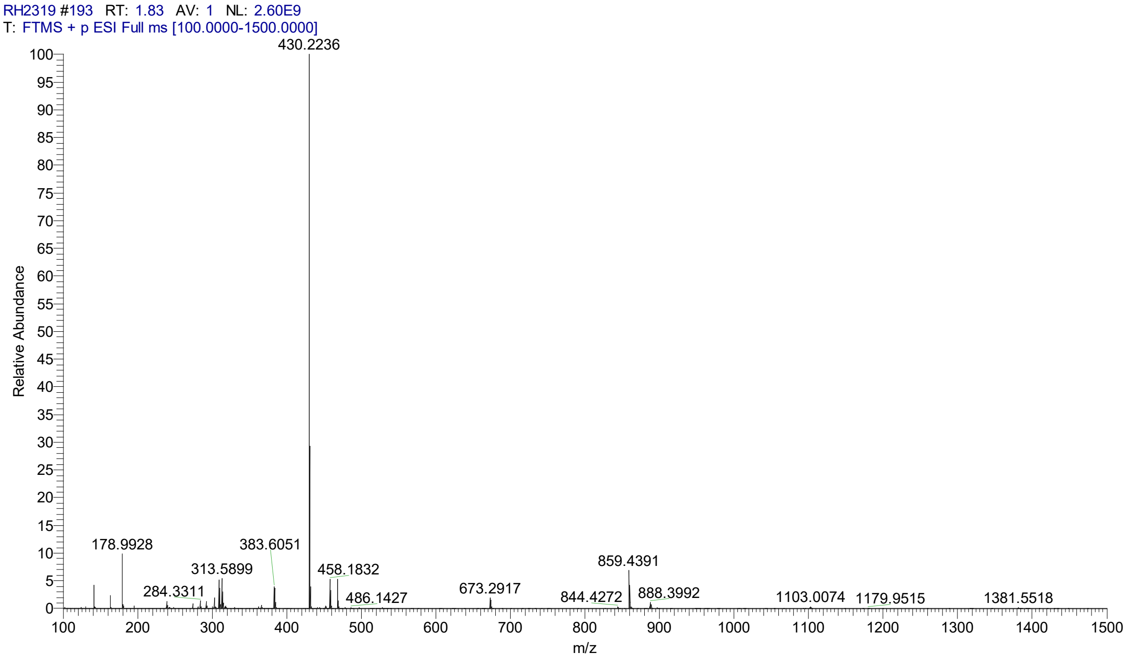

HR-MS Spectrum of compound **3b**

(S)-N-(2-(1H-imidazol-1-yl)ethyl)-3-(1H-indol-3-yl)-2-((S)-2-phenylpropanamido)propanamide (**3c**)

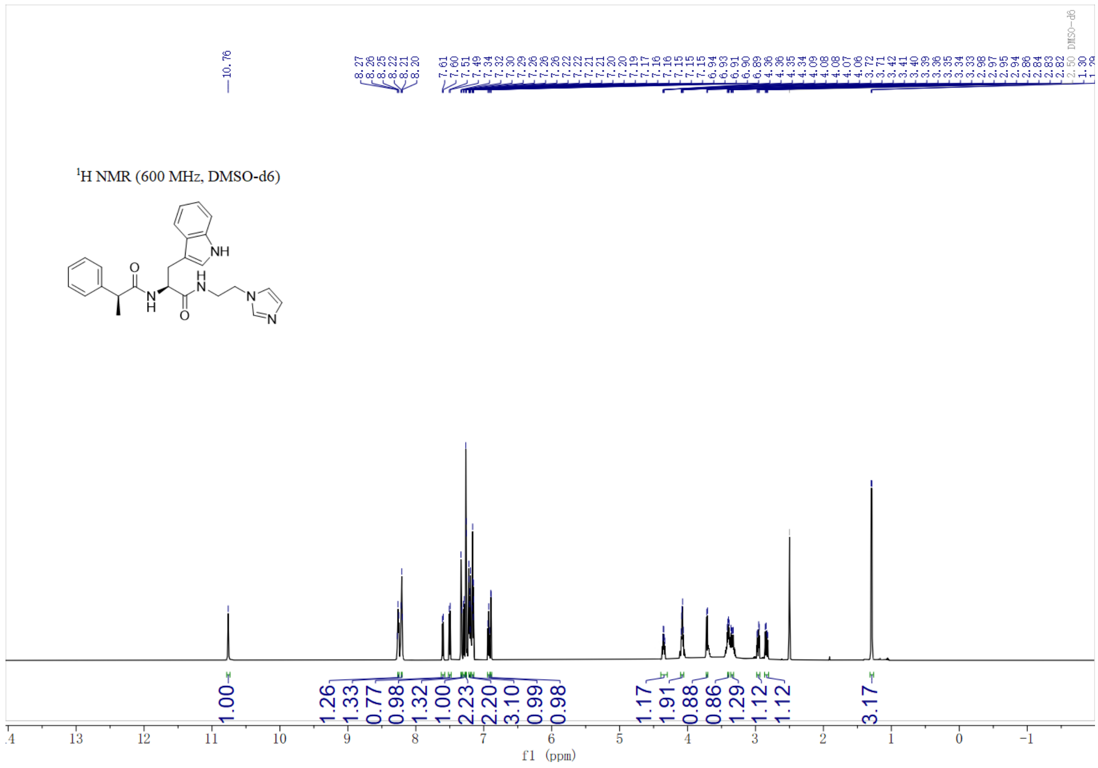

^{1}H NMR Spectrum of compound **3c**

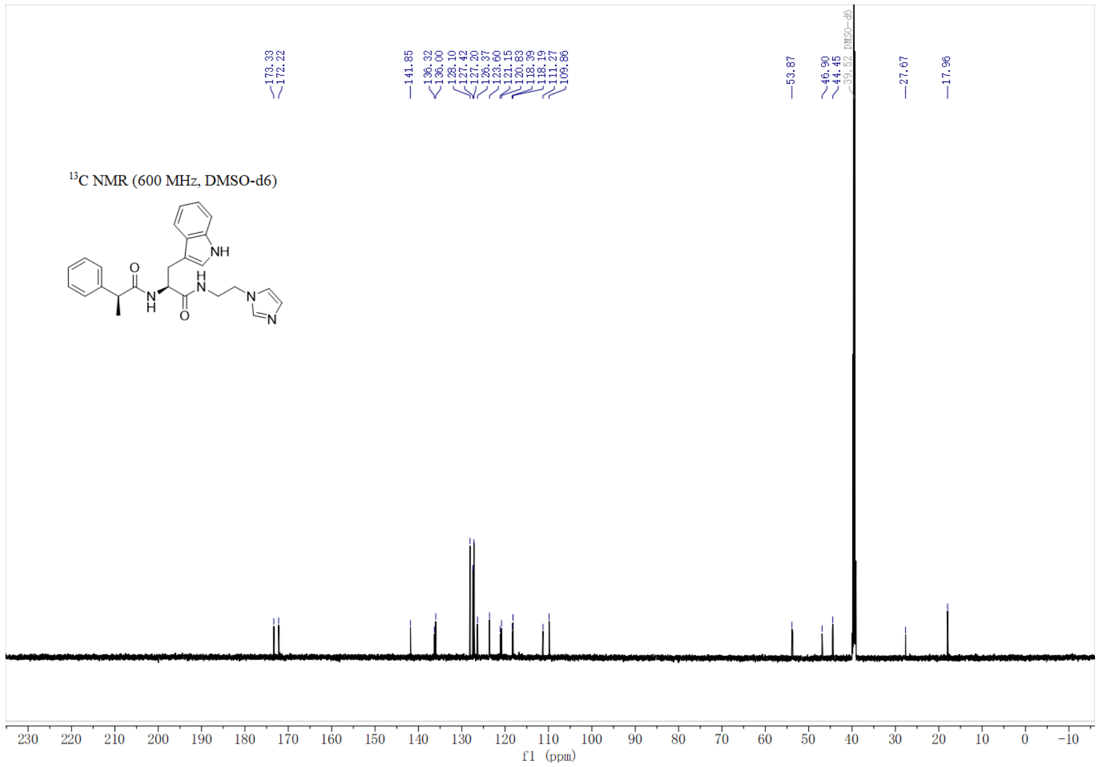

^{13}C NMR Spectrum of compound **3c**

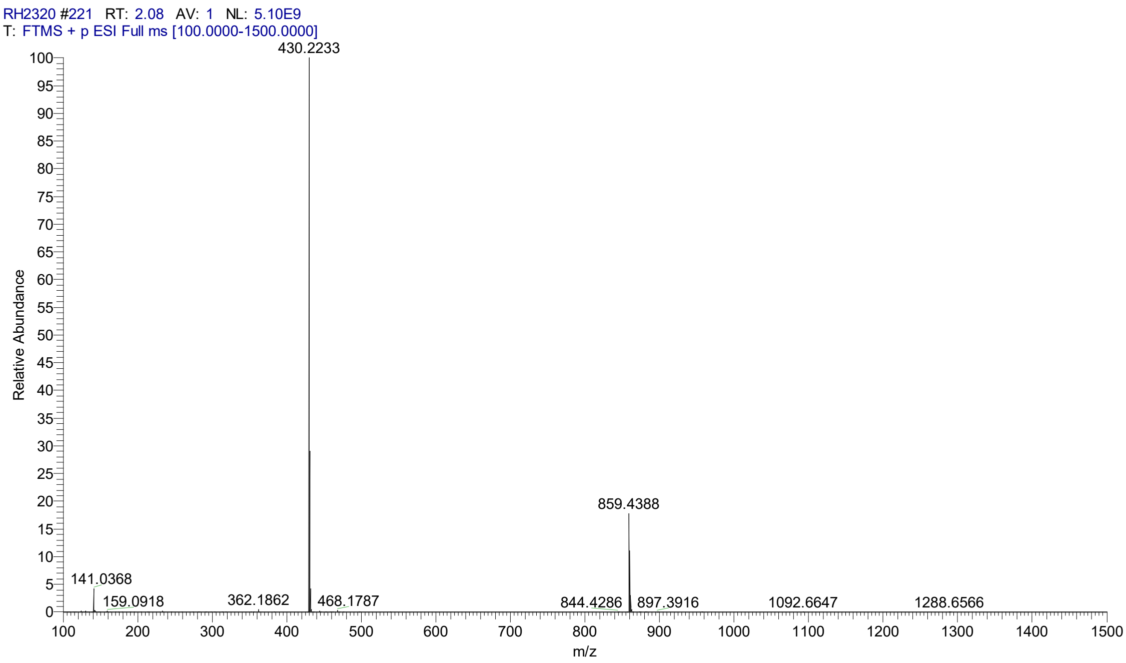

HR-MS Spectrum of compound **3c**

(2S)-N-(2-(1H-imidazol-1-yl)ethyl)-3-(1H-indol-3-yl)-2-(2-(4-nitrophenyl)propanamido)propanamide (**3d**)

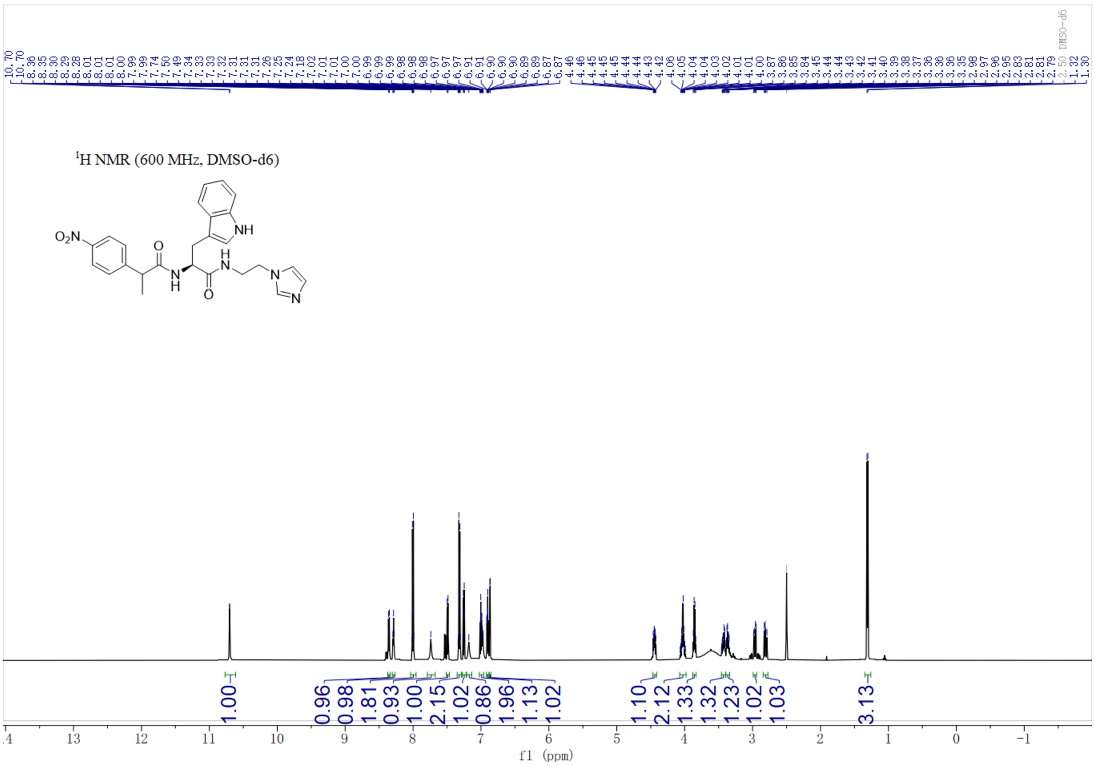

^{1}H NMR Spectrum of compound **3d**

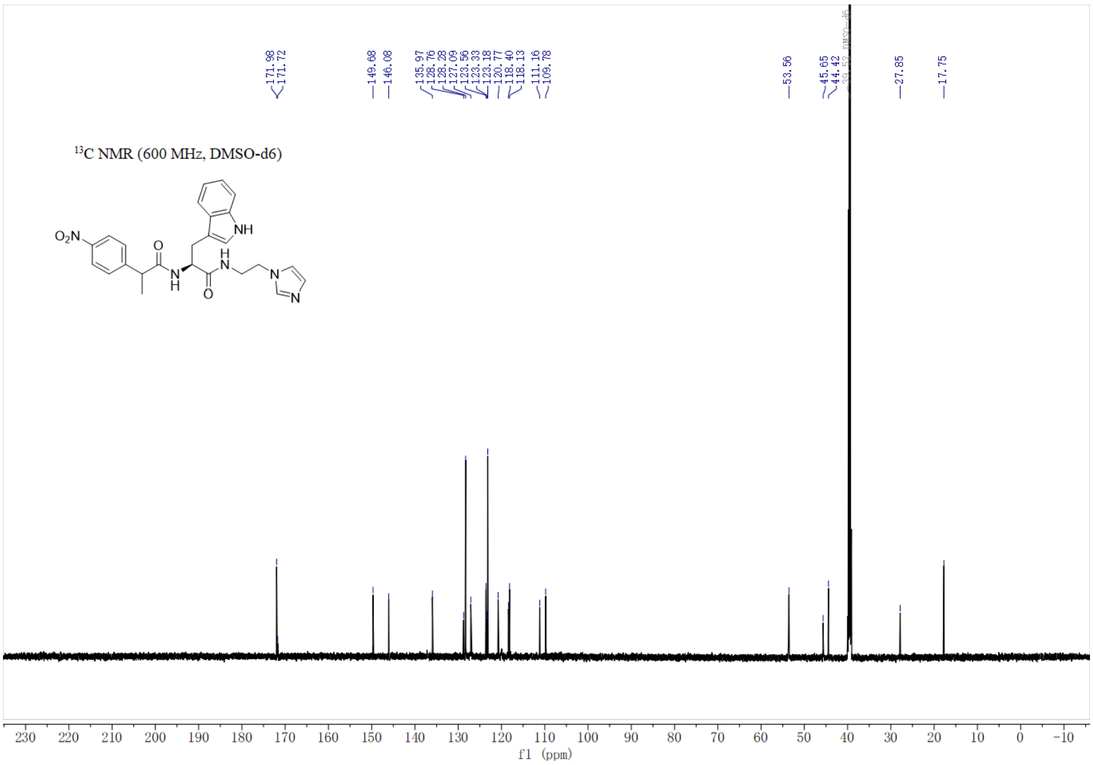

^{13}C NMR Spectrum of compound **3d**

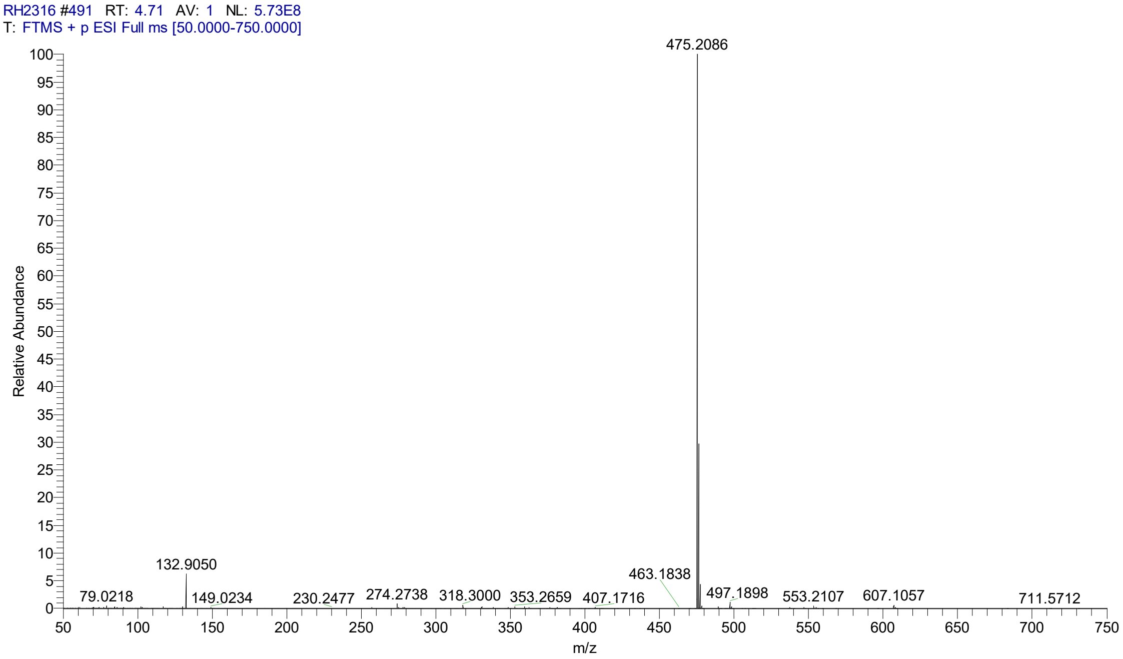

HR-MS Spectrum of compound **3d**

(2S)-N-(2-(1H-imidazol-1-yl)ethyl)-2-(2-(3-chlorophenyl)propanamido)-3-(1H-indol-3-yl)propanamide (**3e**)

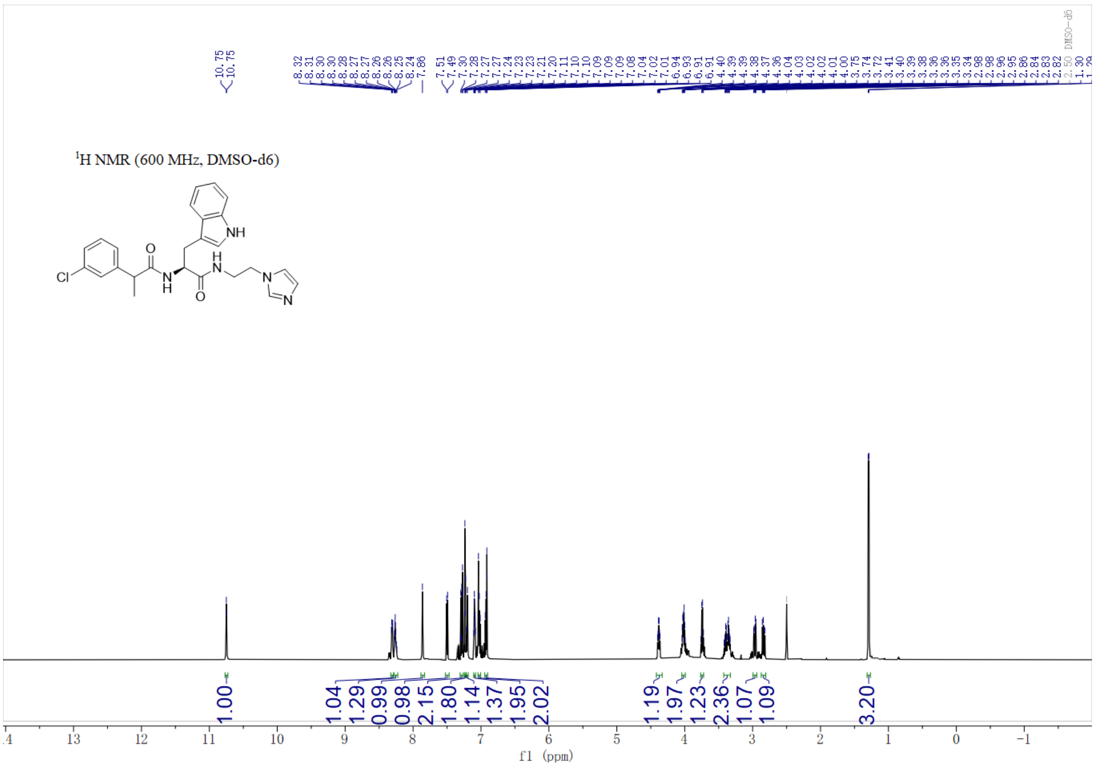

^{1}H NMR Spectrum of compound **3e**

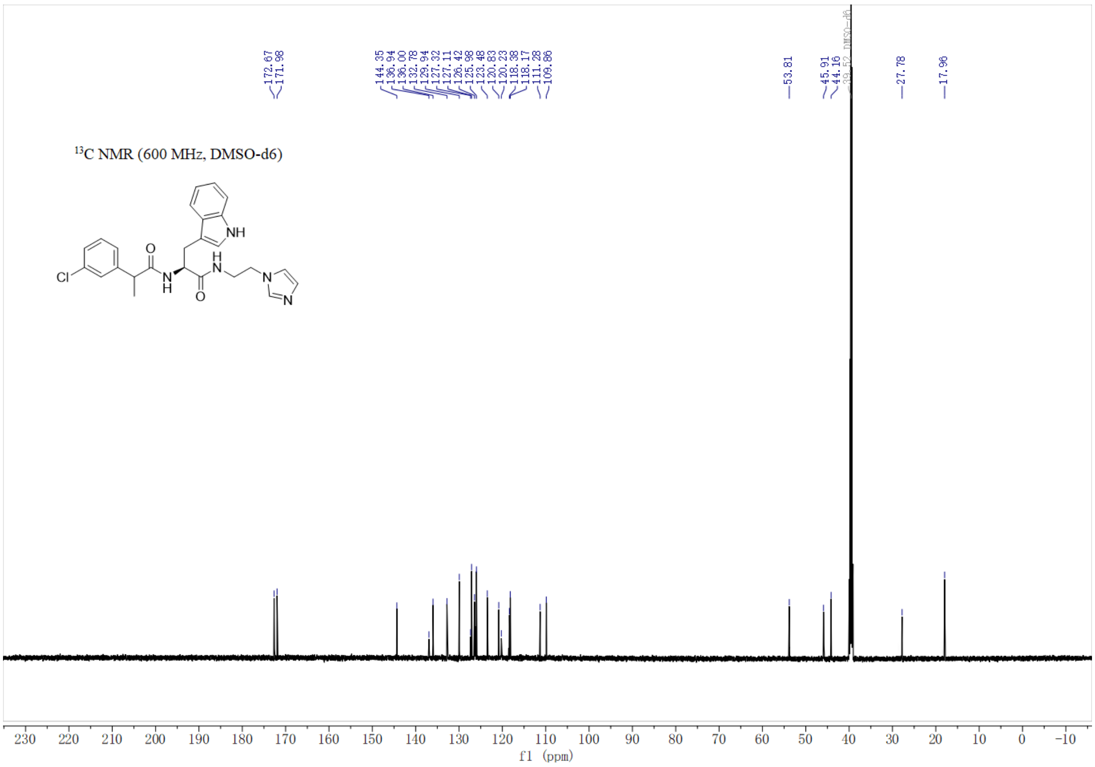

^{13}C NMR Spectrum of compound **3e**

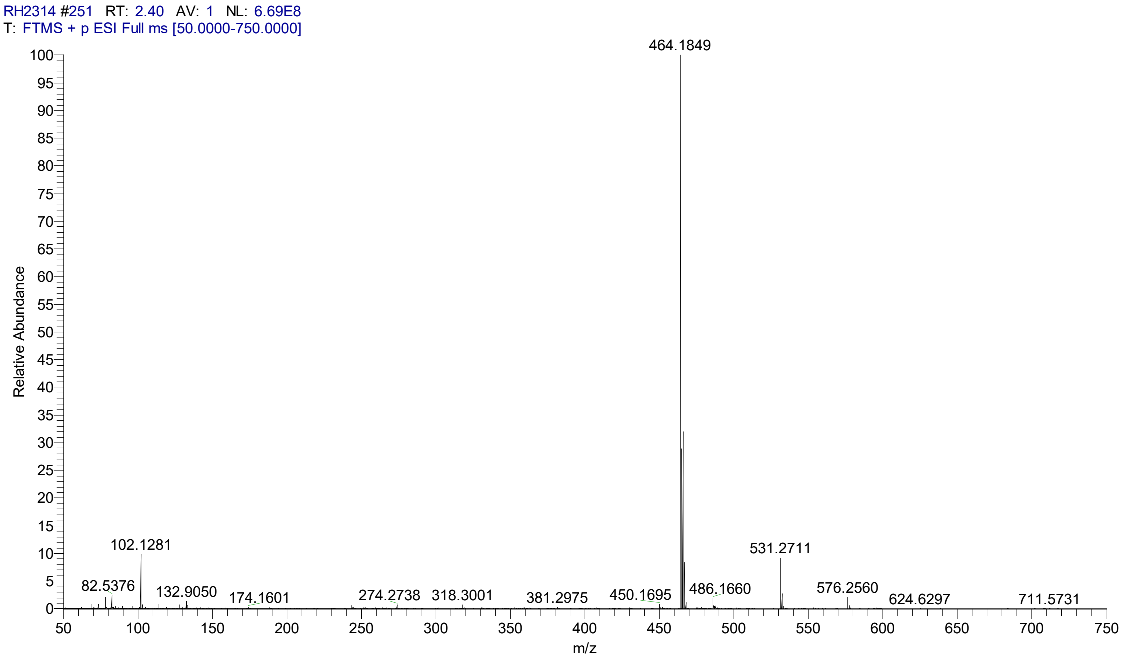

HR-MS Spectrum of compound **3e**

(2S)-N-(2-(1H-imidazol-1-yl)ethyl)-3-(1H-indol-3-yl)-2-(2-(p-tolyl)propanamido)propanamide (**3f**)

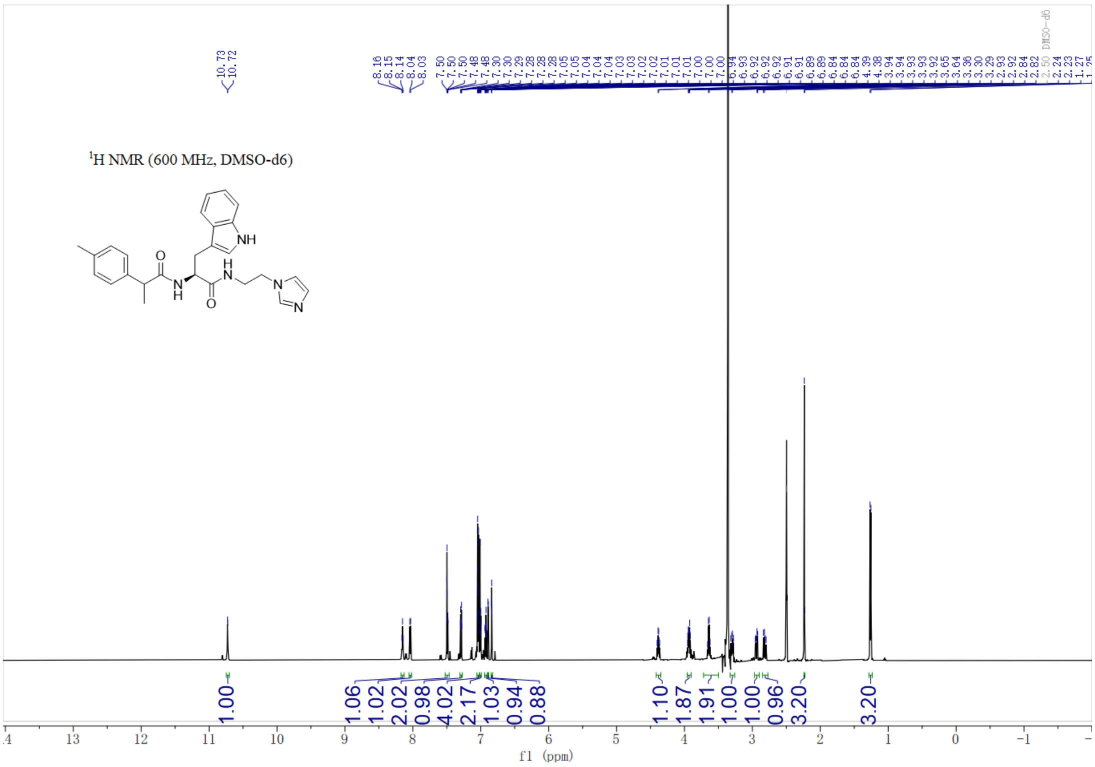

^{1}H NMR Spectrum of compound **3f**

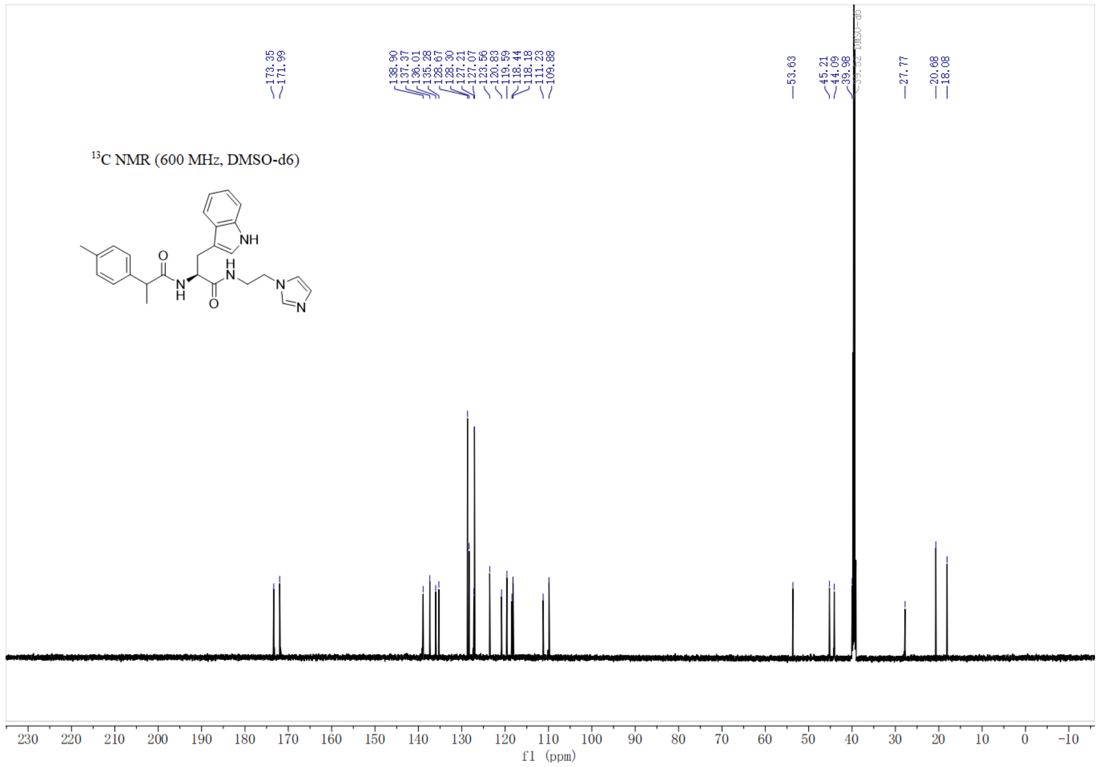

^{13}C NMR Spectrum of compound **3f**

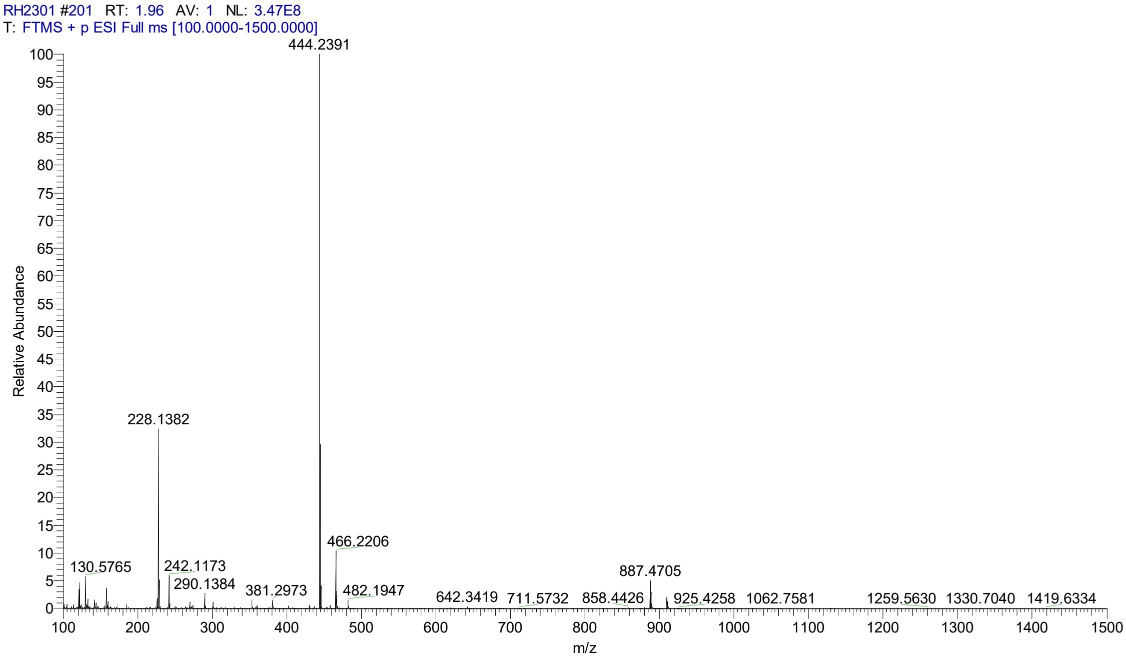

HR-MS Spectrum of compound **3f**

(2S)-N-(2-(1H-imidazol-1-yl)ethyl)-3-(1H-indol-3-yl)-2-(2-(4-methyl-3-nitrophenyl)propanamido)propanamide (**3g**)

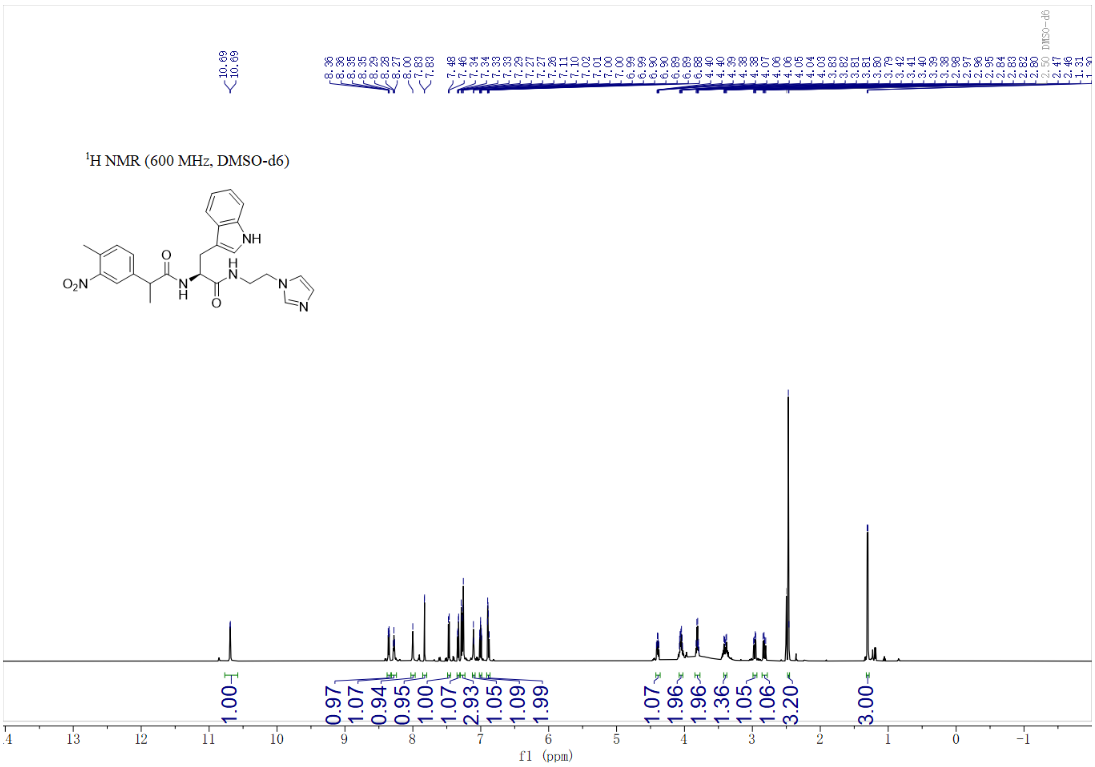

^{1}H NMR Spectrum of compound **3g**

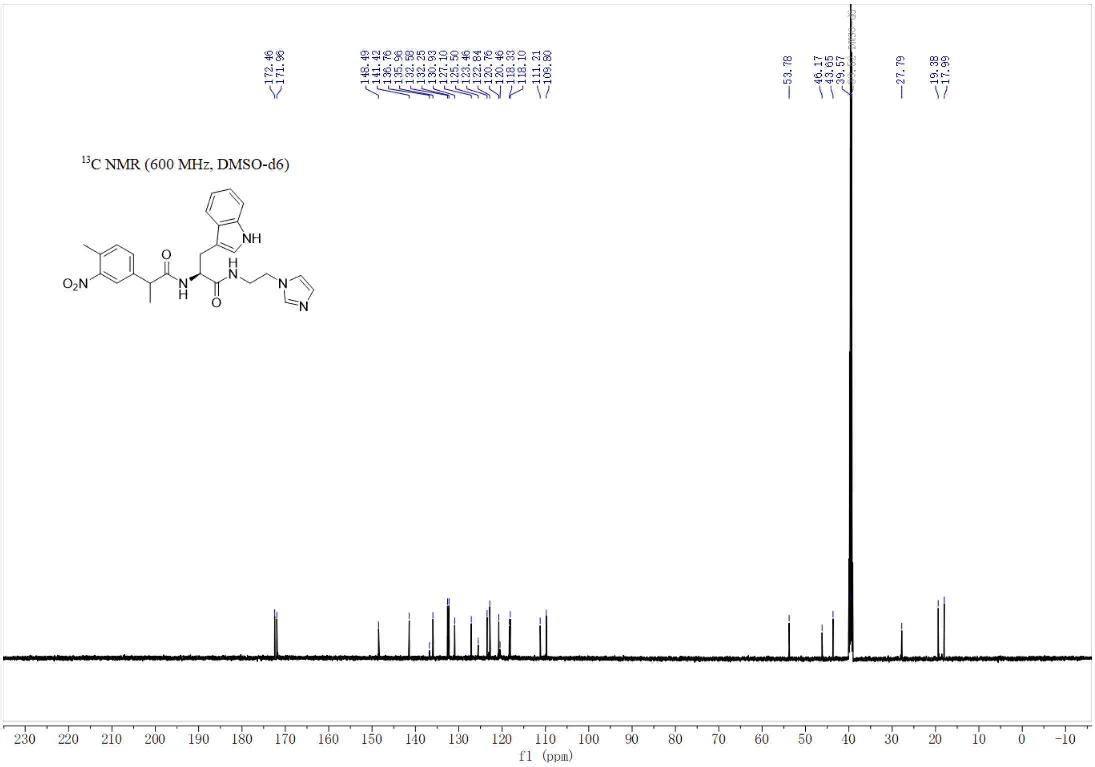

^{13}C NMR Spectrum of compound **3g**

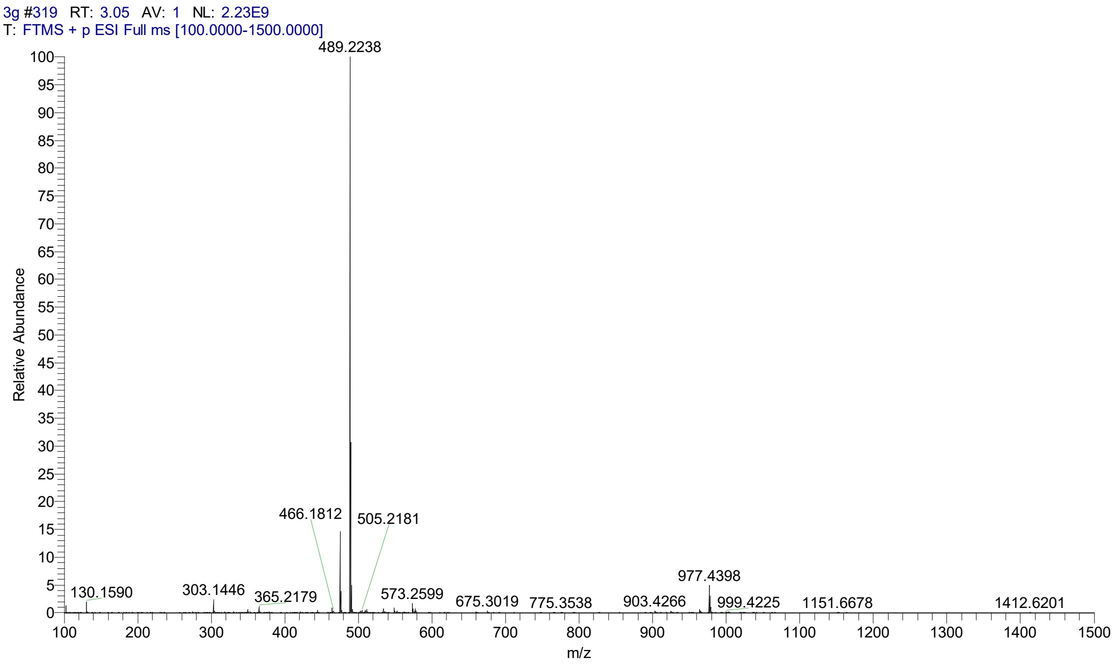

HR-MS Spectrum of compound **3g**

(2S)-N-(2-(1H-imidazol-1-yl)ethyl)-3-(1H-indol-3-yl)-2-(2-(4-methyl-3,5-dinitrophenyl)propanamido)propanamide (**3h**)

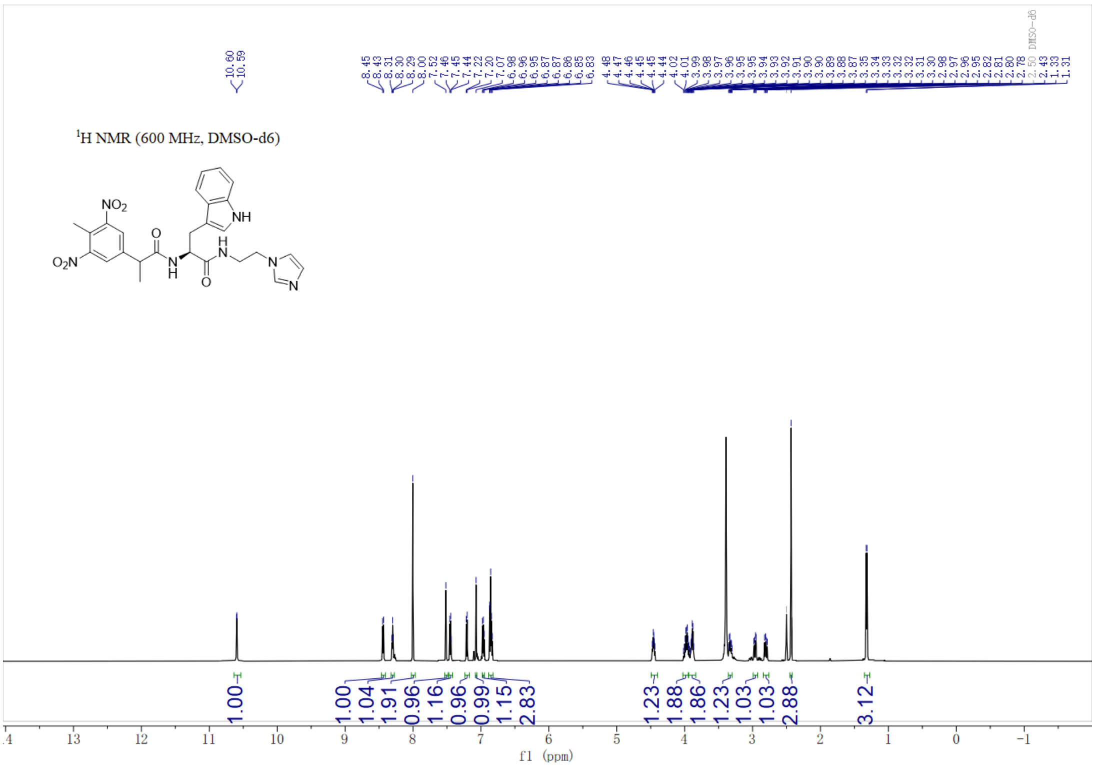

^{1}H NMR Spectrum of compound **3h**

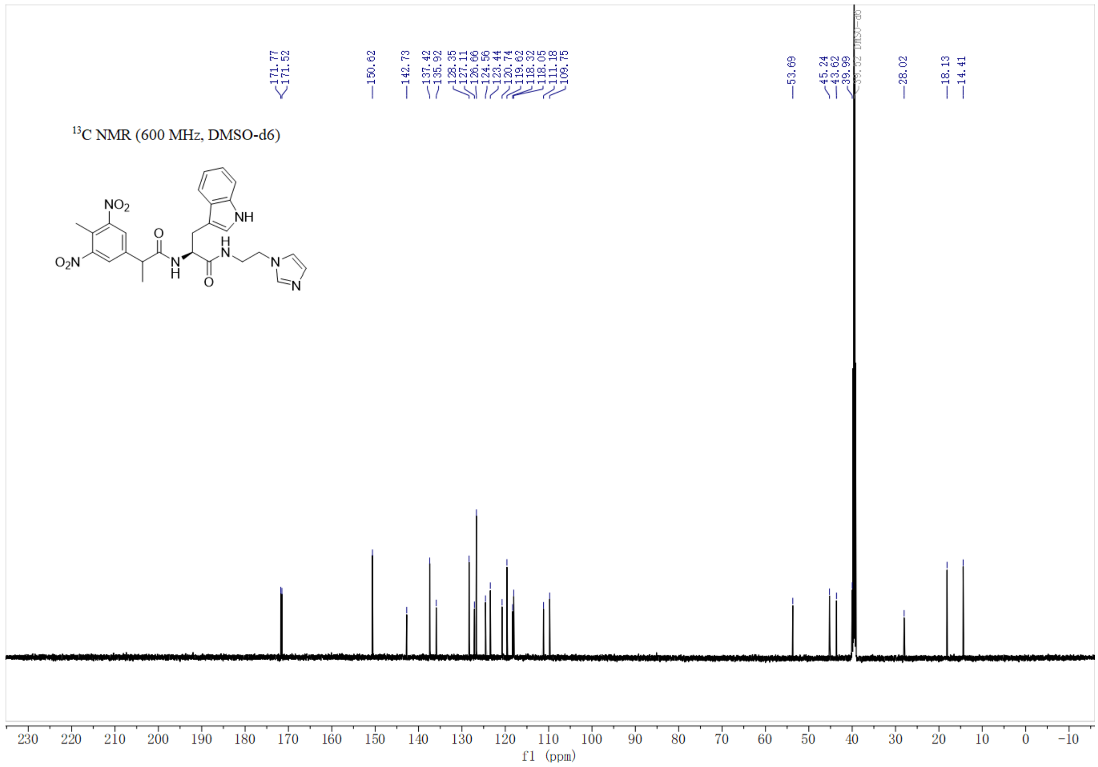

^{13}C NMR Spectrum of compound **3h**

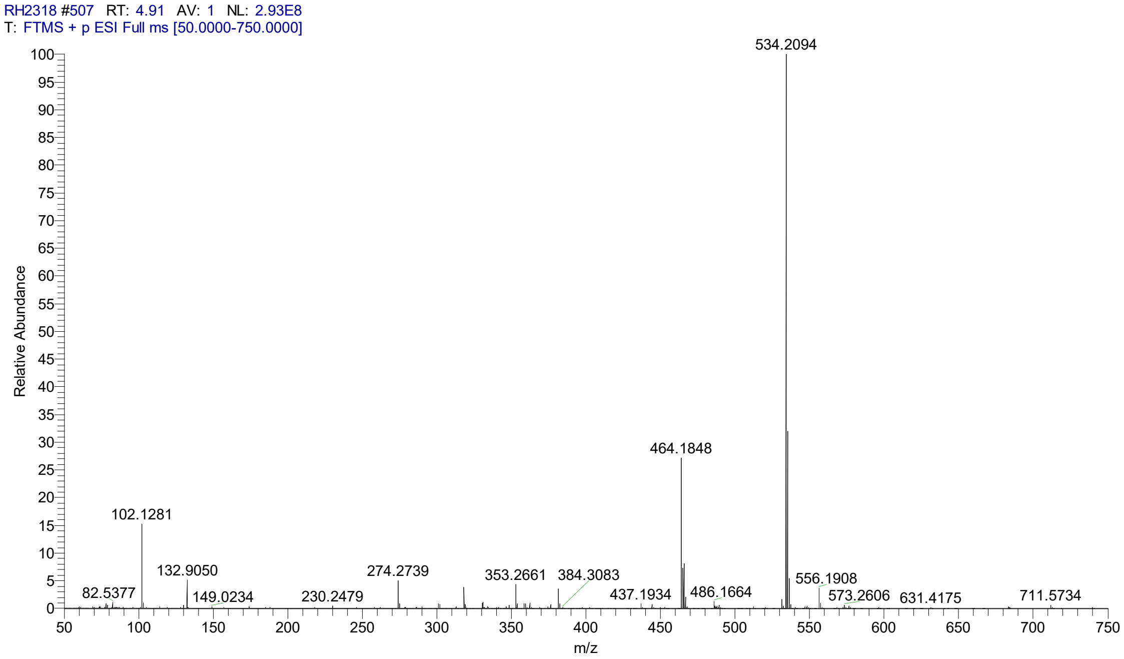

HR-MS Spectrum of compound **3h**

(2S)-N-(2-(1H-imidazol-1-yl)ethyl)-3-(1H-indol-3-yl)-2-(2-(4-isobutylphenyl)propanamido)propanamide (**3i**)

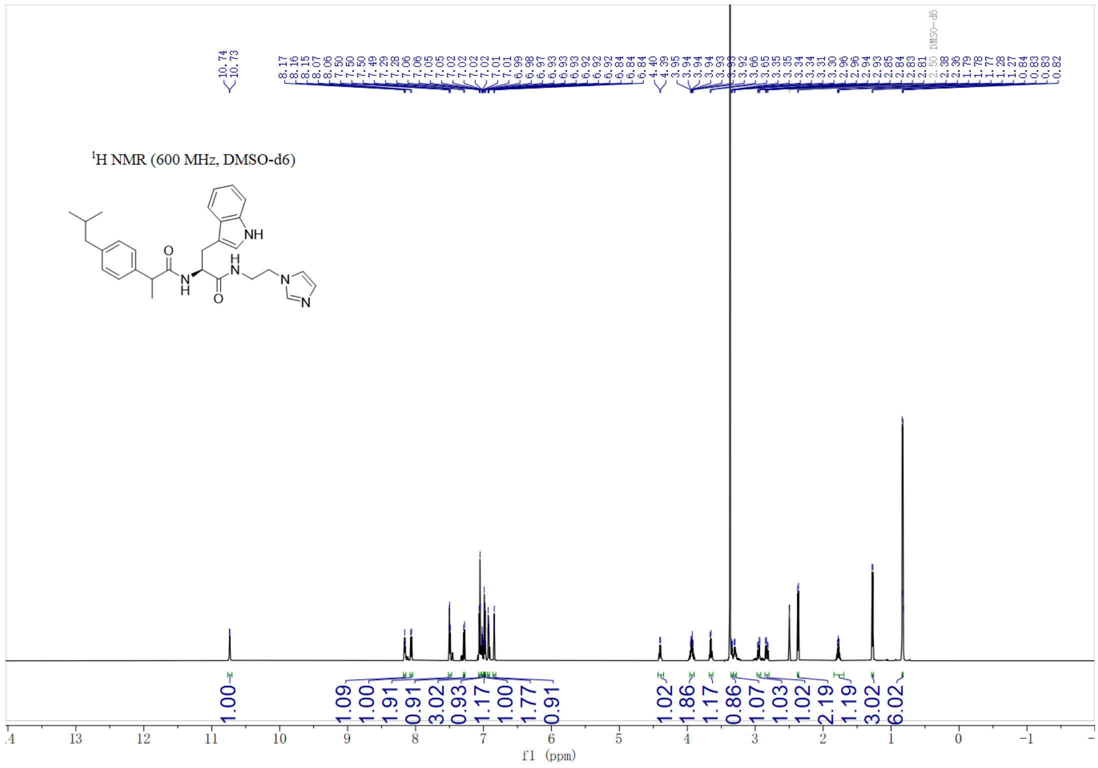

^{1}H NMR Spectrum of compound **3i**

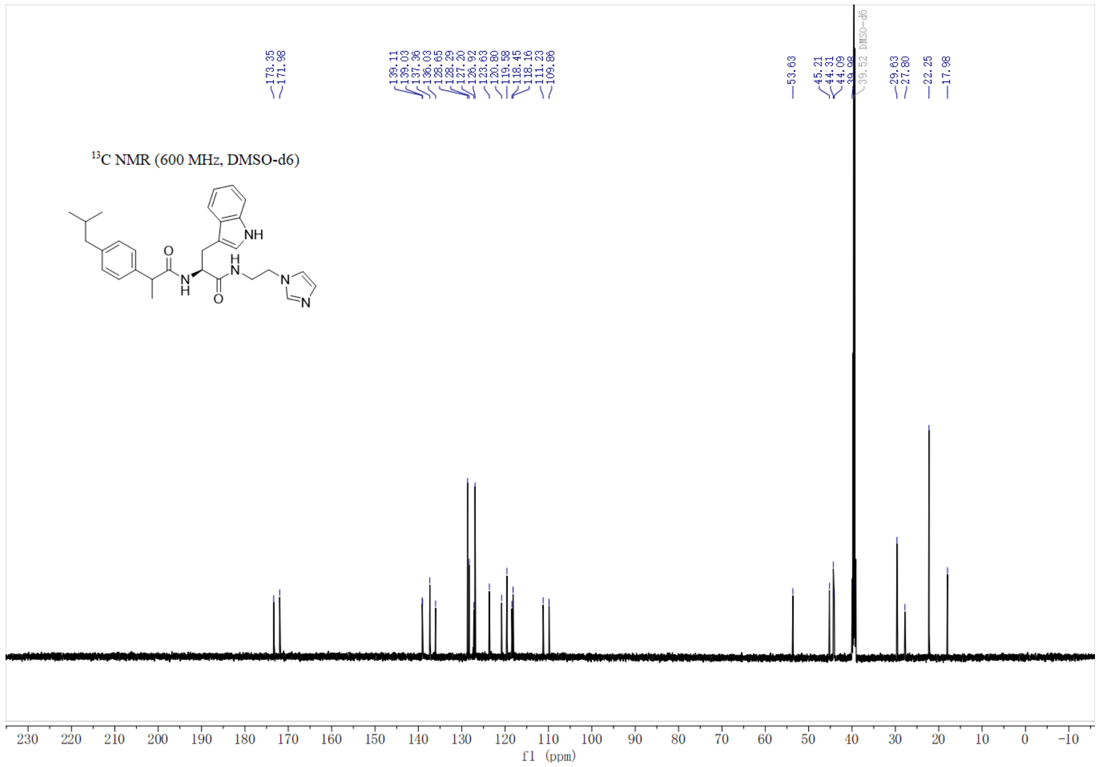

^{13}C NMR Spectrum of compound **3i**

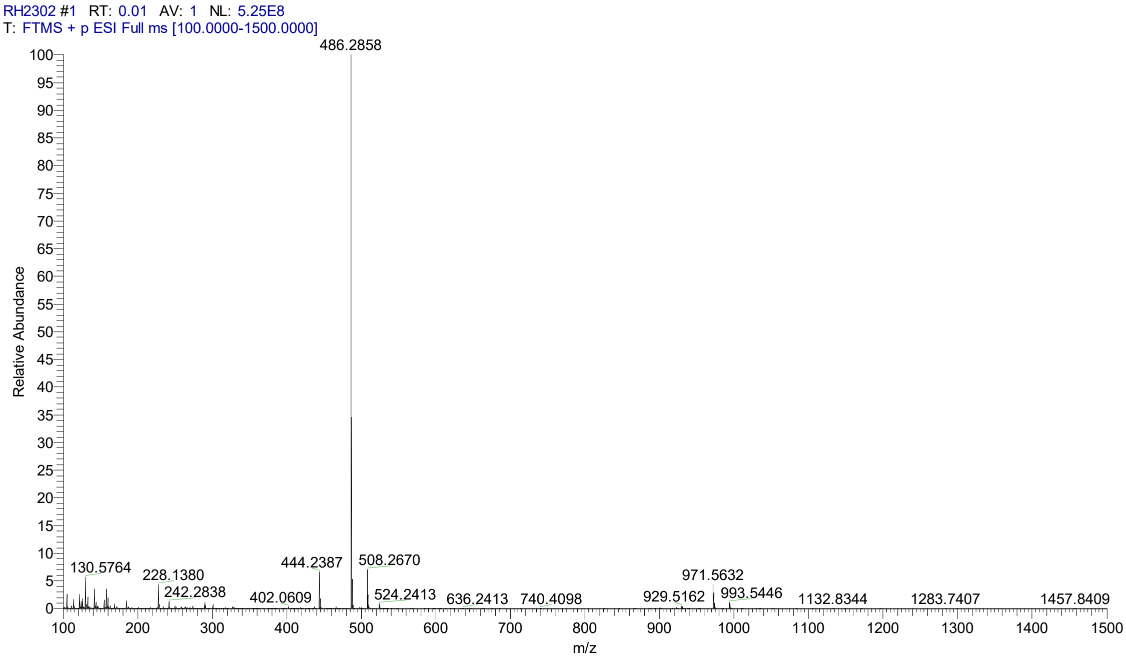

HR-MS Spectrum of compound **3i**

(S)-N-(2-(1H-imidazol-1-yl)ethyl)-3-(1H-indol-3-yl)-2-((R)-2-(4-isobutylphenyl)propanamido)propanamide (**3j**)

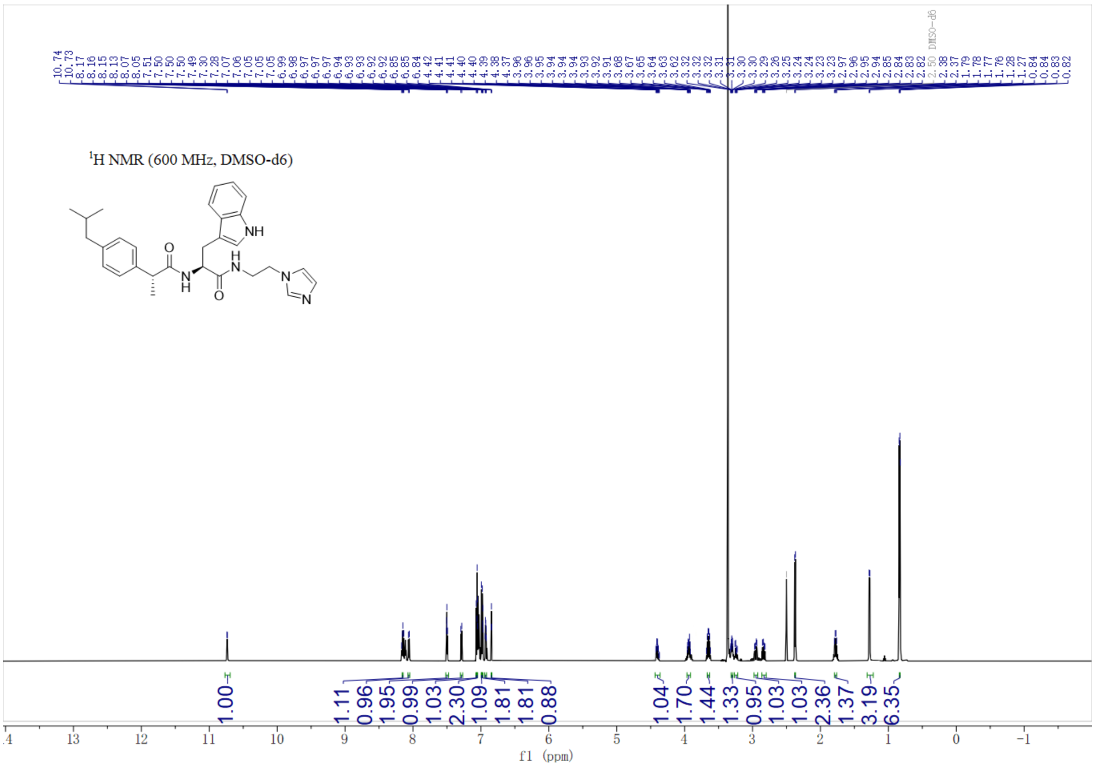

^{1}H NMR Spectrum of compound **3j**

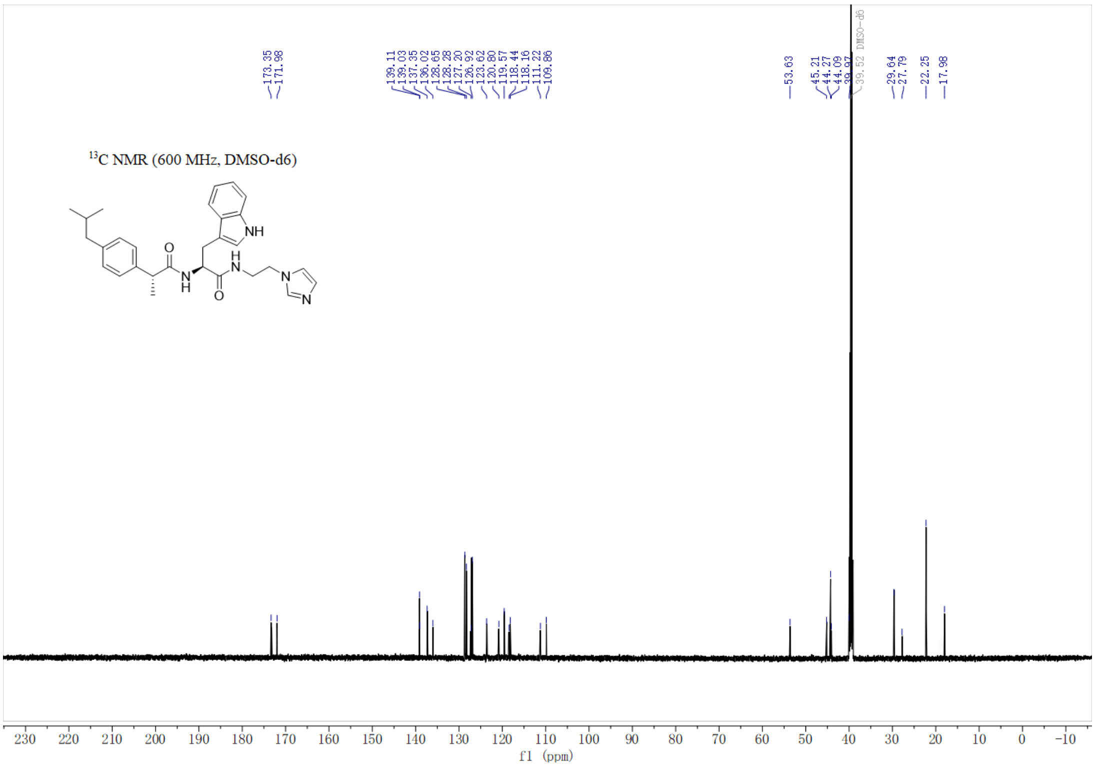

^{13}C NMR Spectrum of compound **3j**

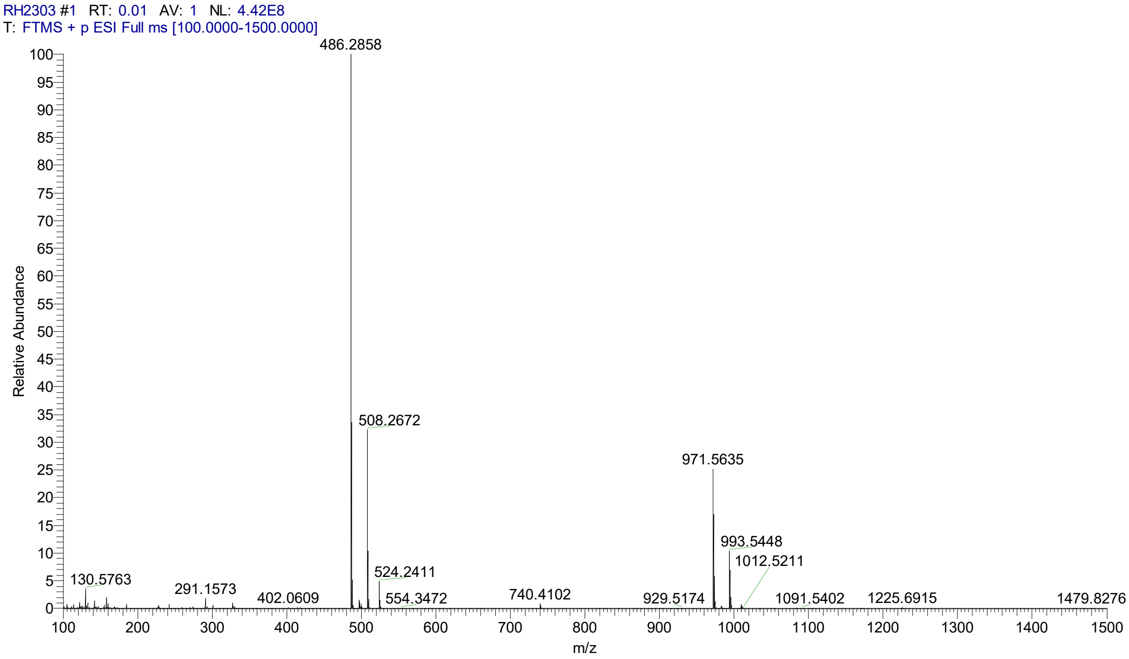

HR-MS Spectrum of compound **3j**

(2S)-N-(2-(1H-imidazol-1-yl)ethyl)-3-(1H-indol-3-yl)-2-(2-(4-((2-oxocyclopentyl)methyl)phenyl)propanamido)propanamide (**3k**)

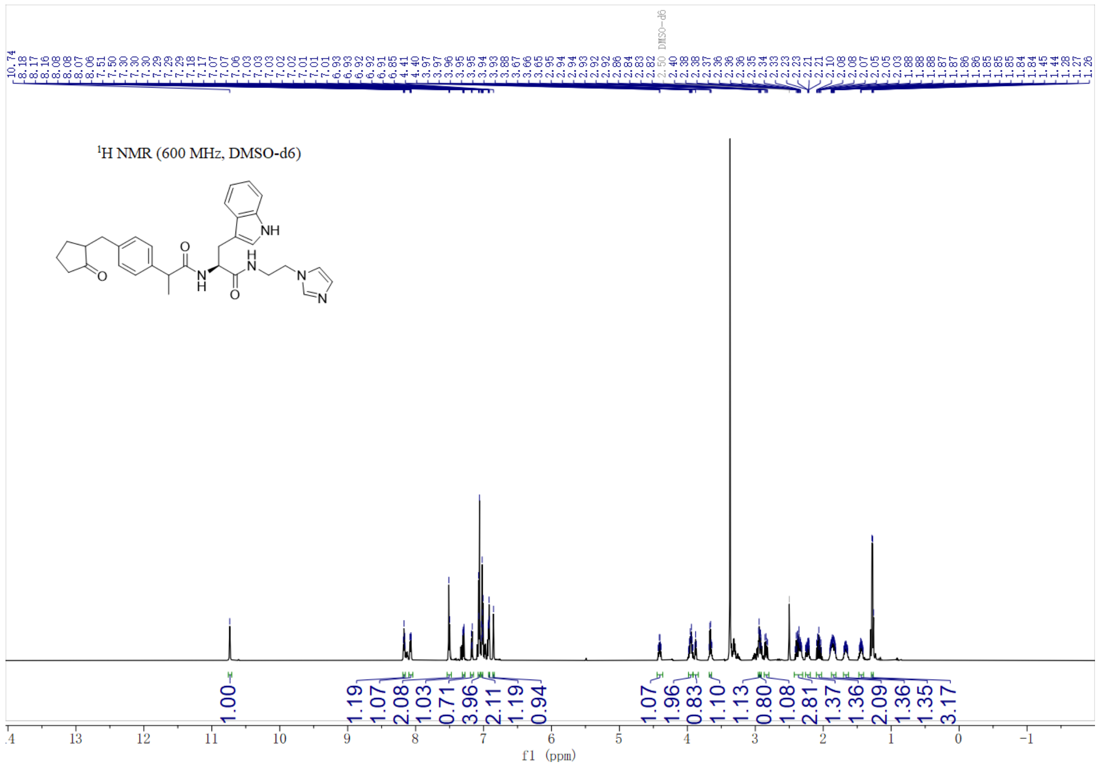

^{1}H NMR Spectrum of compound **3k**

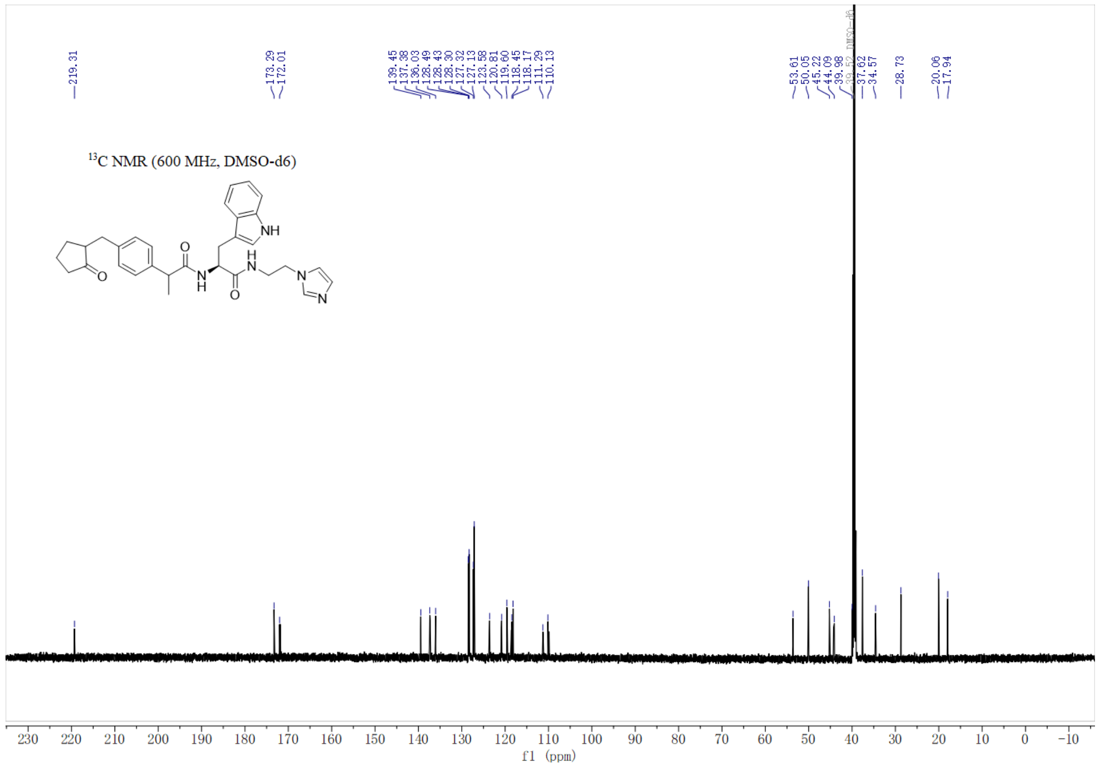

^{13}C NMR Spectrum of compound **3k**

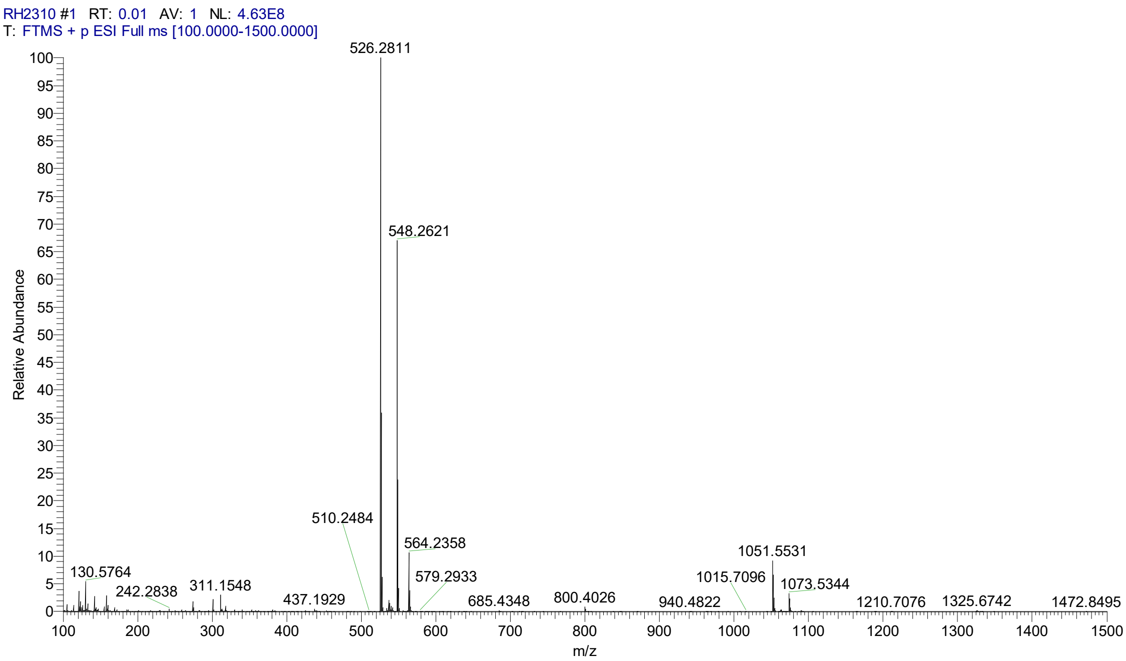

HR-MS Spectrum of compound **3k**

(2S)-N-(2-(1H-imidazol-1-yl)ethyl)-2-(2-(3-benzoylphenyl)propanamido)-3-(1H-indol-3-yl)propanamide (**3l**)

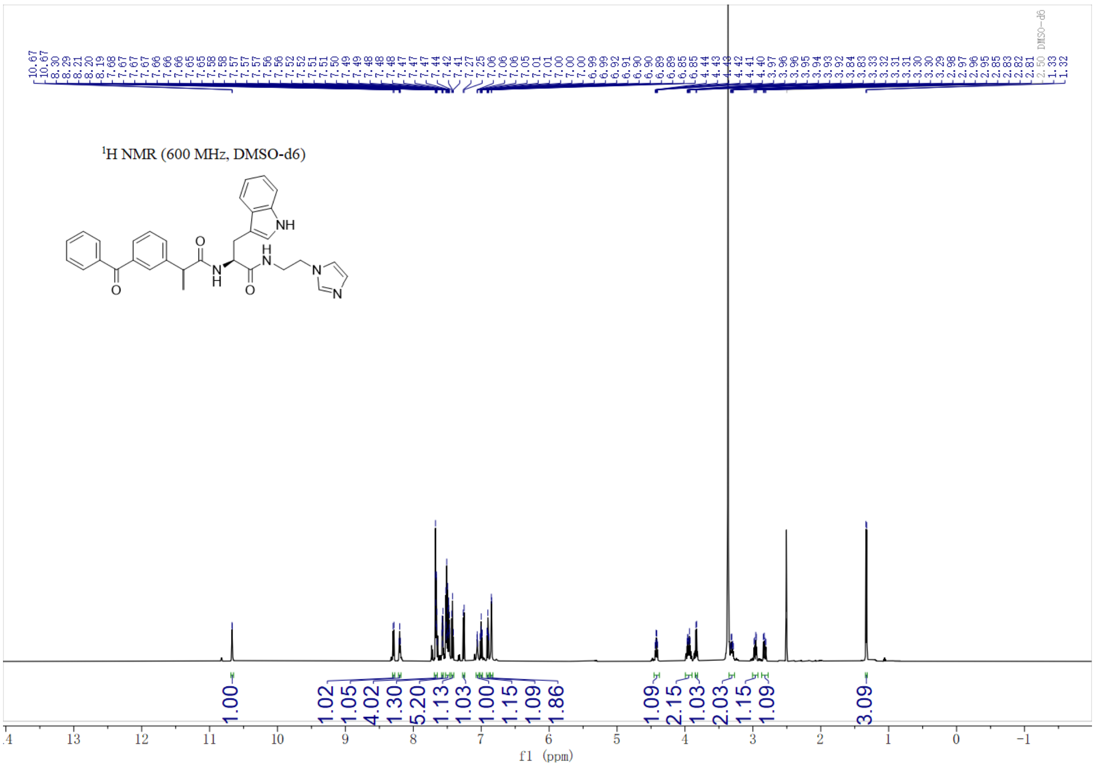

^{1}H NMR Spectrum of compound **3l**

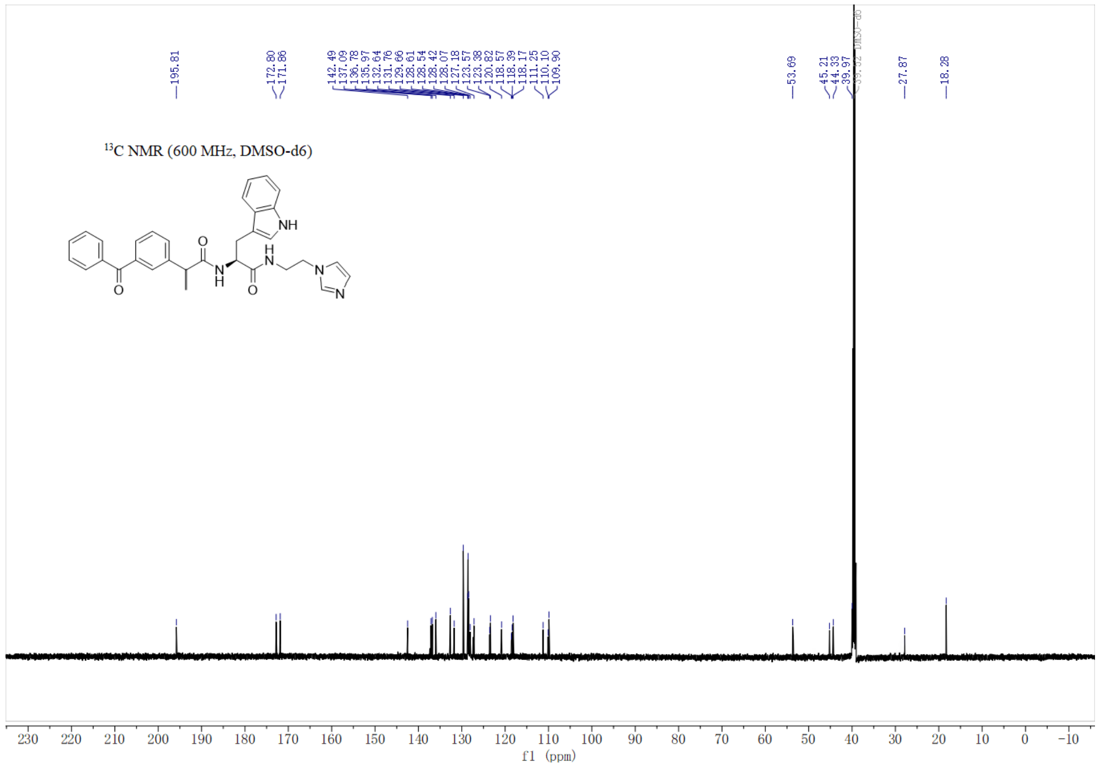

^{13}C NMR Spectrum of compound **3l**

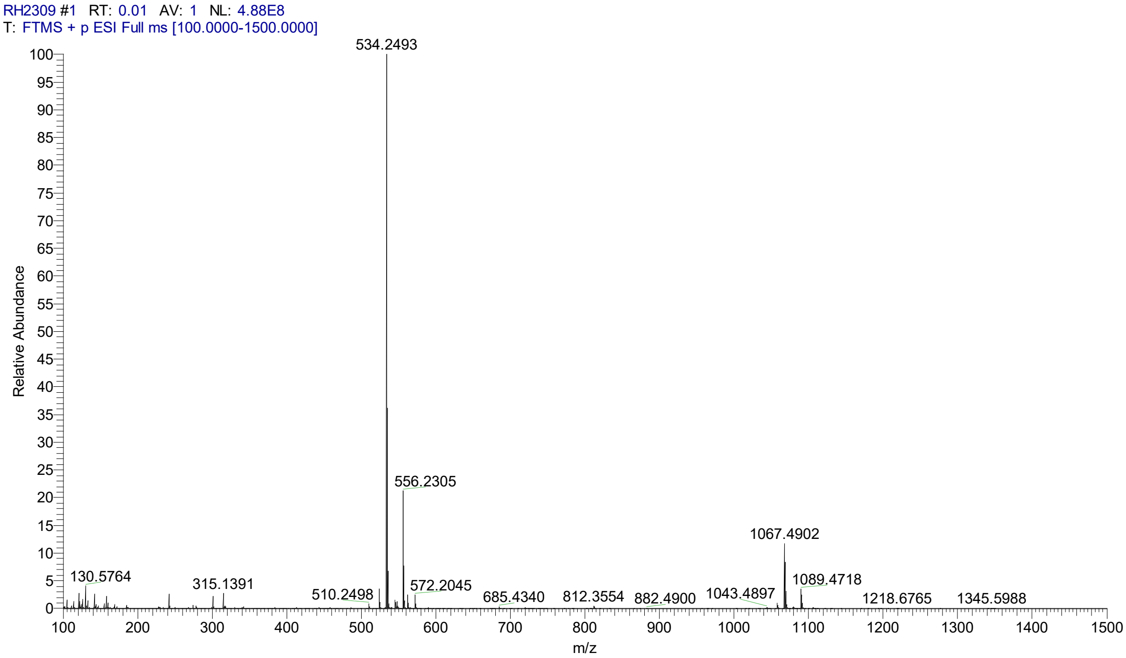

HR-MS Spectrum of compound **3l**

(S)-N-(2-(1H-imidazol-1-yl)ethyl)-3-(1H-indol-3-yl)-2-((S)-2-(6-methoxynaphthalen-2-yl)propanamido)propanamide (**3m**)

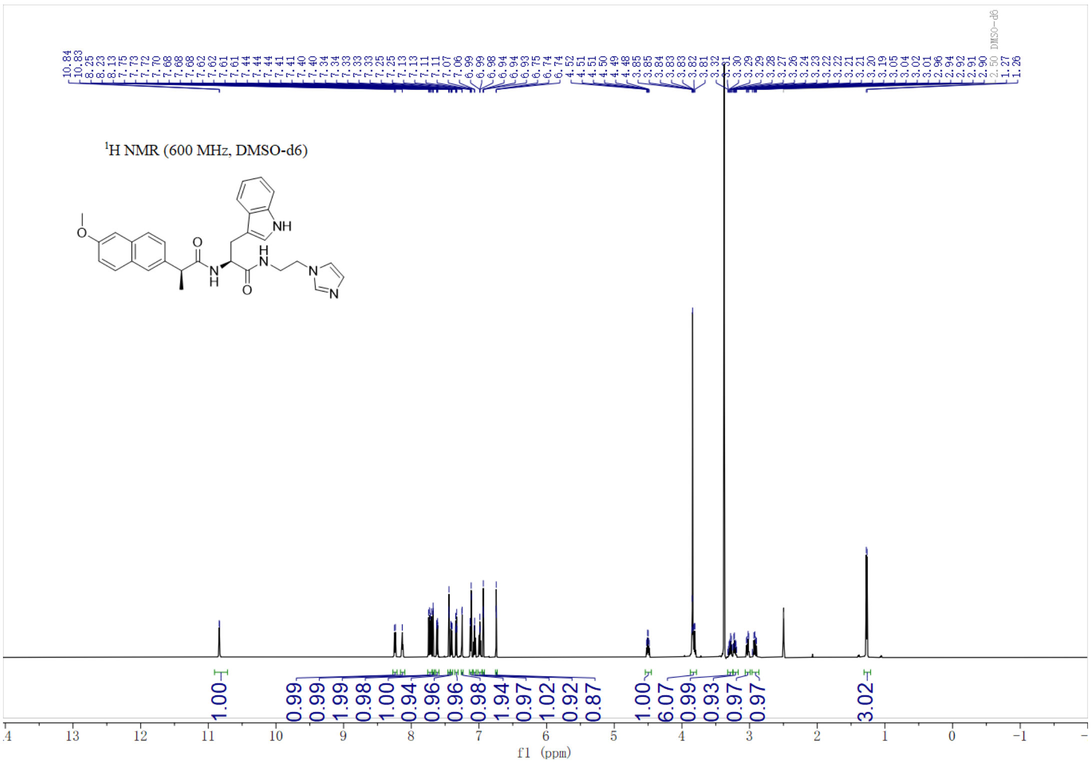

^{1}H NMR Spectrum of compound **3m**

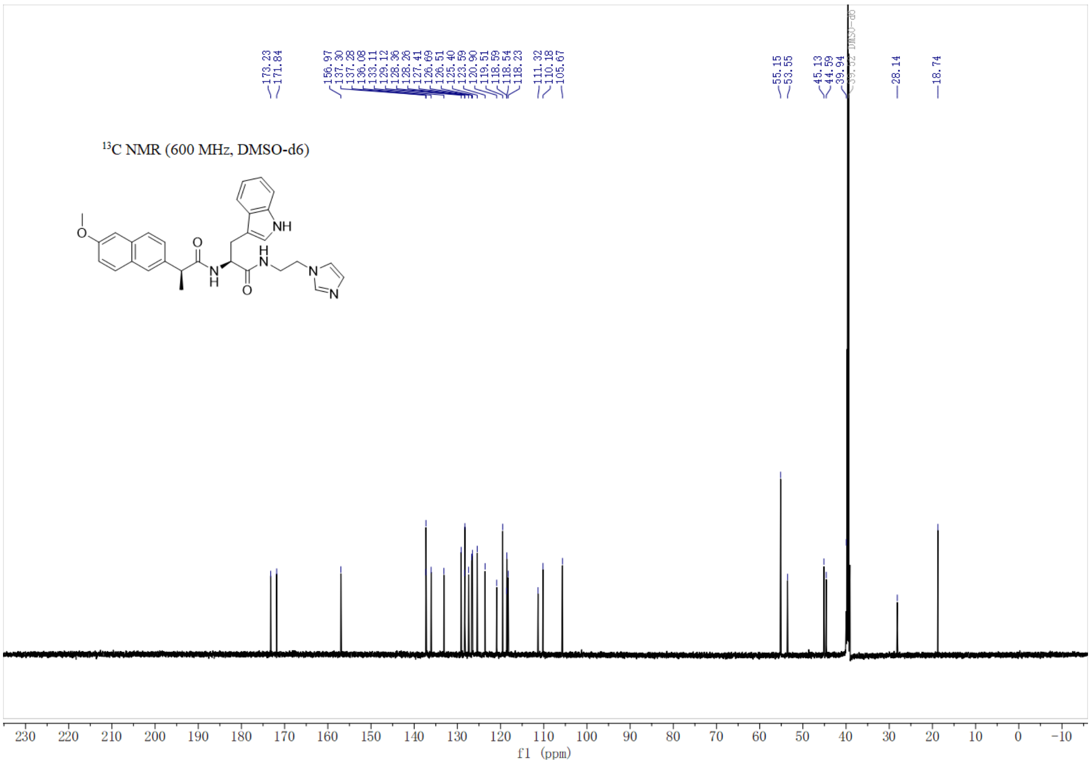

^{13}C NMR Spectrum of compound **3m**

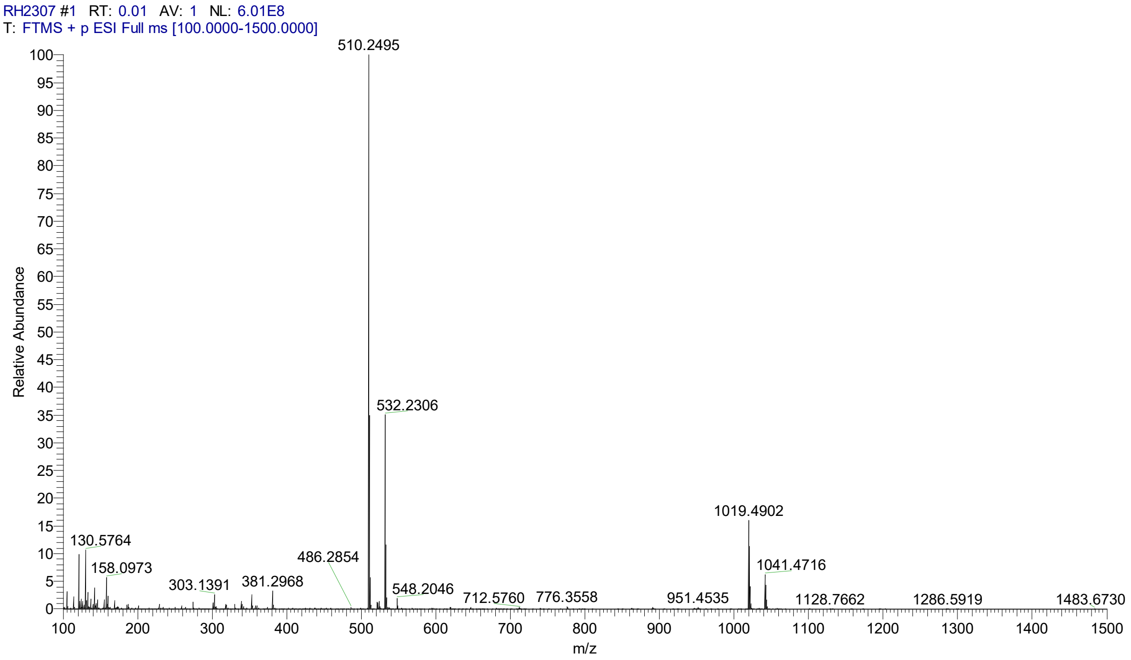

HR-MS Spectrum of compound **3m**

(2S)-N-(2-(1H-imidazol-1-yl)ethyl)-2-(2-(2-fluoro-[1,1'-biphenyl]-4-yl)propanamido)-3-(1H-indol-3-yl)propanamide (**3n**)

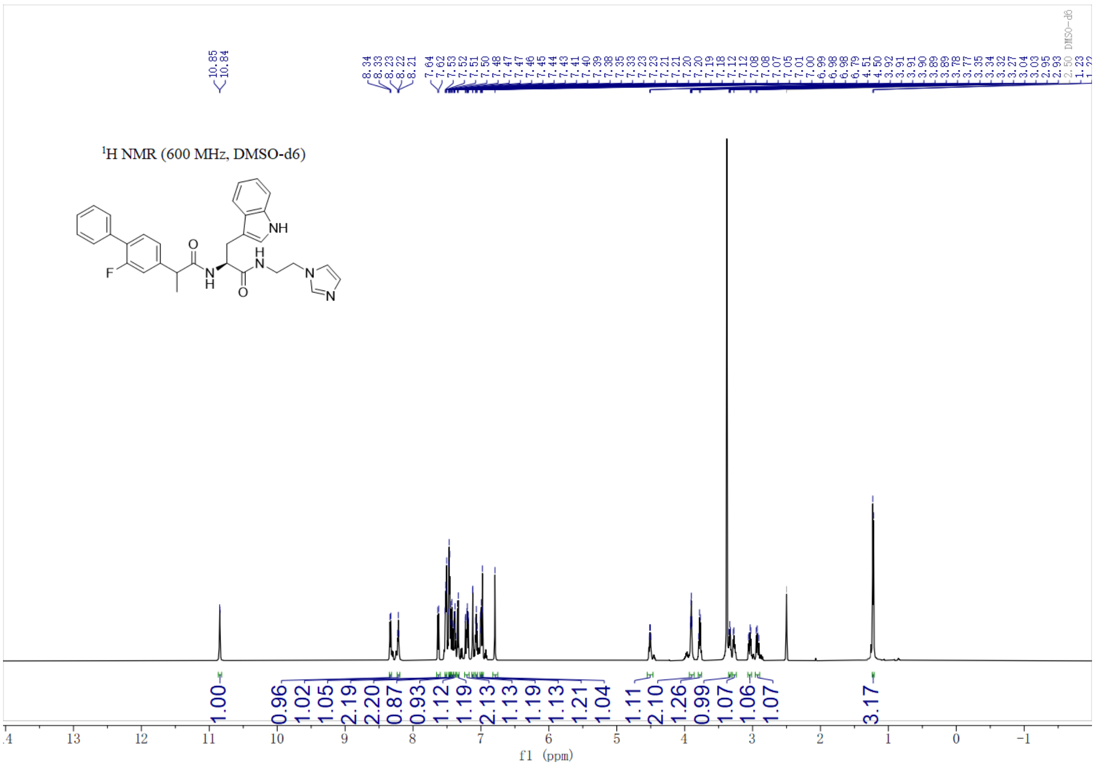

^{1}H NMR Spectrum of compound **3n**

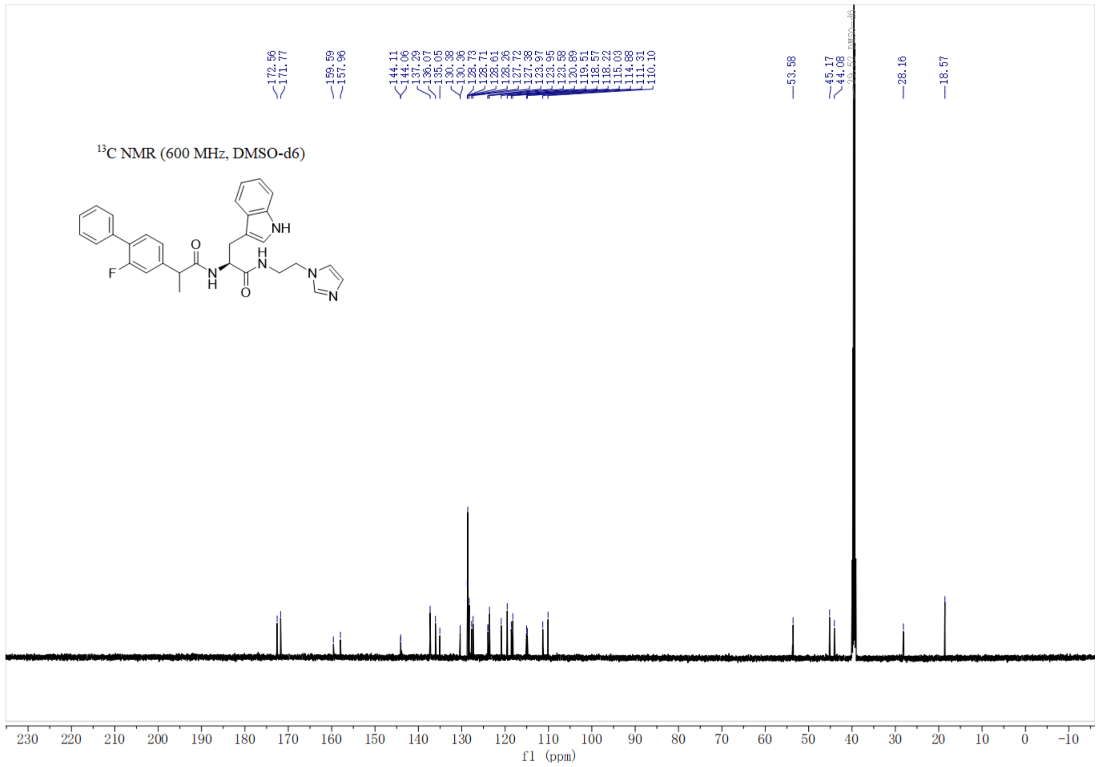

^{13}C NMR Spectrum of compound **3n**

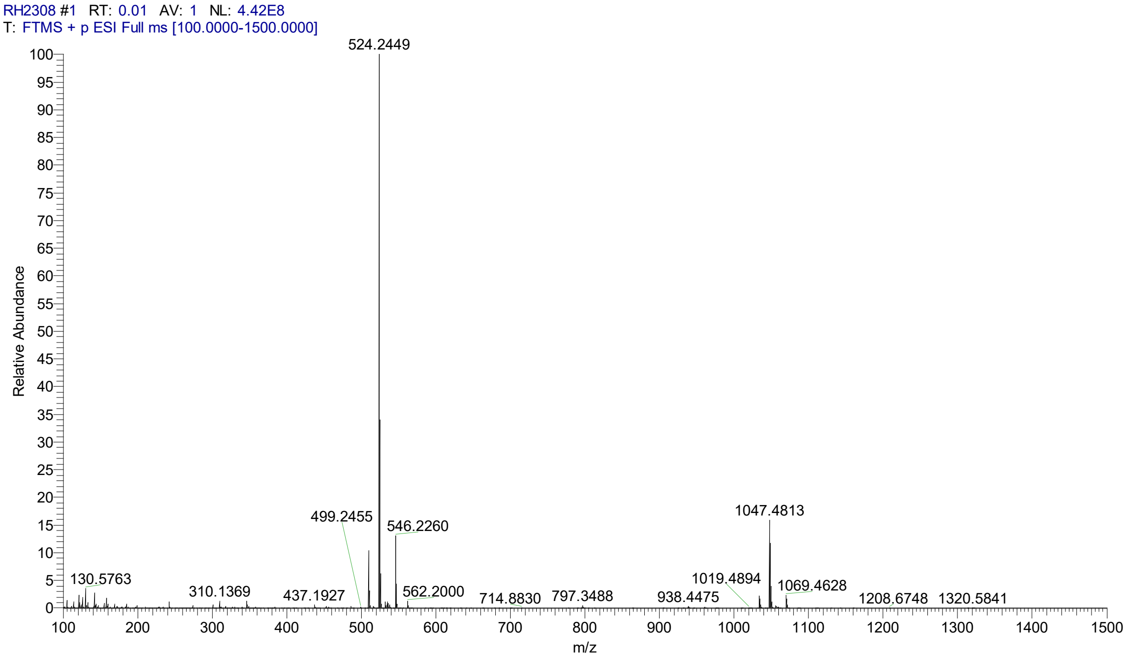

HR-MS Spectrum of compound **3n**

(2S)-N-(2-(1H-imidazol-1-yl)ethyl)-2-(2-(6-chloro-9H-carbazol-2-yl)propanamido)-3-(1H-indol-3-yl)propanamide (**3o**)

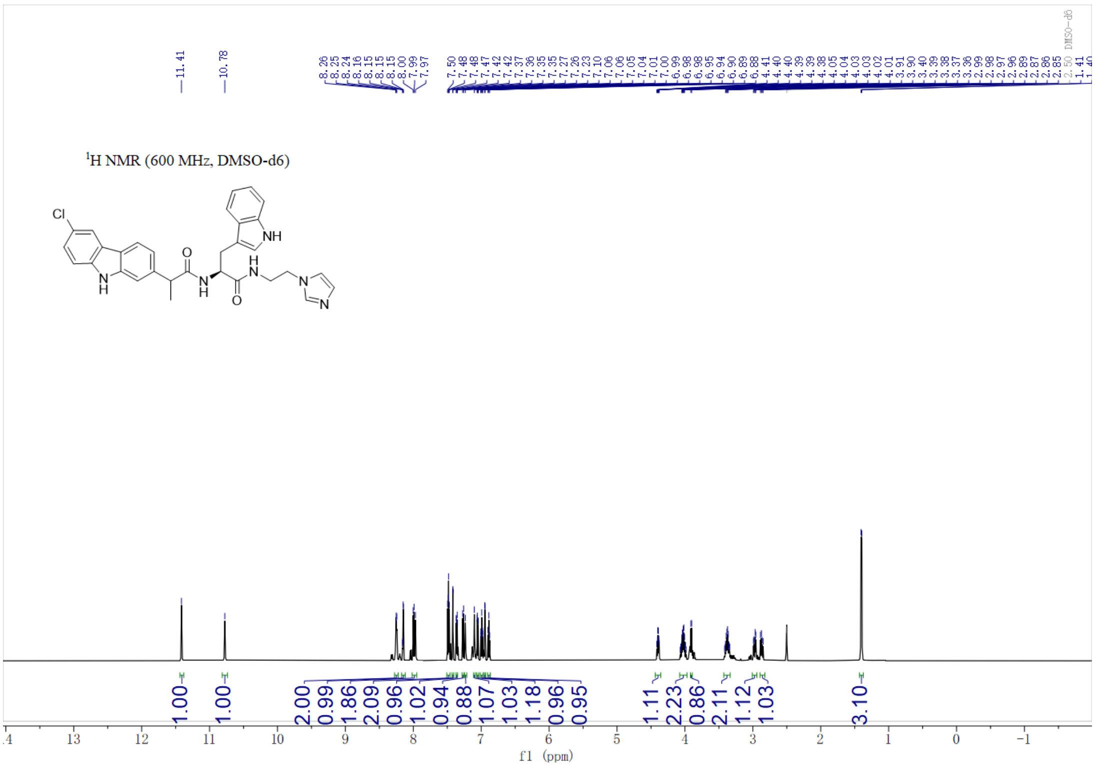

^{1}H NMR Spectrum of compound **3o**

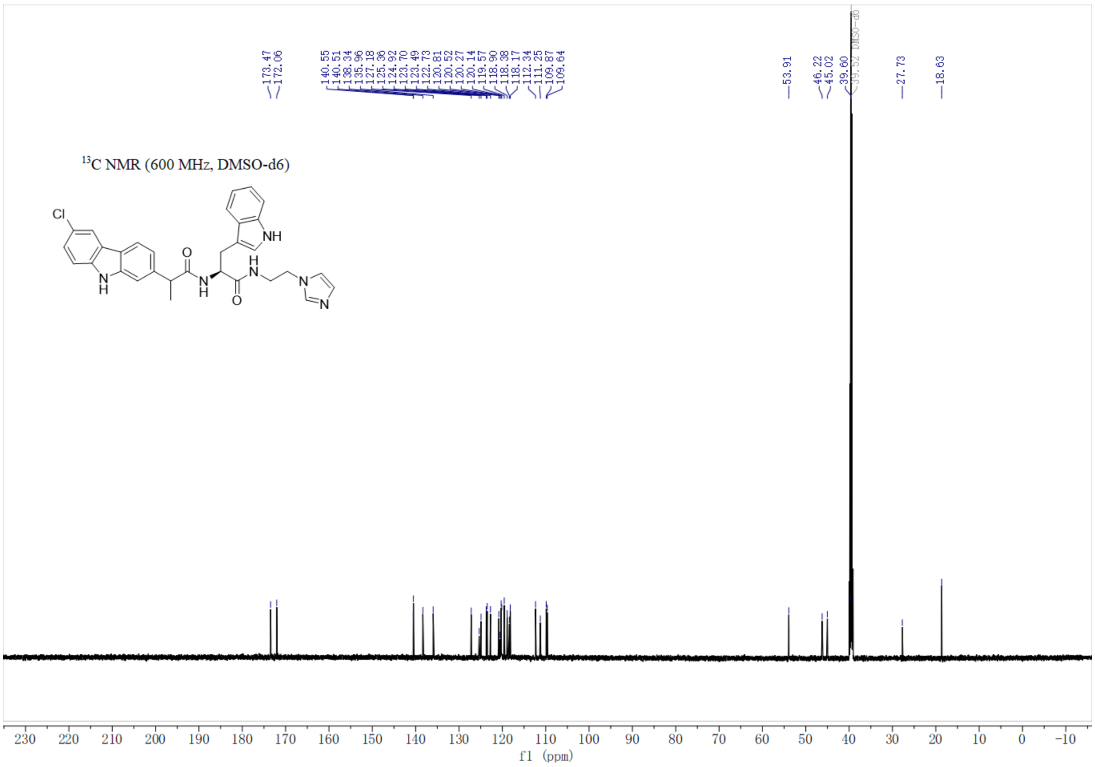

^{13}C NMR Spectrum of compound **3o**

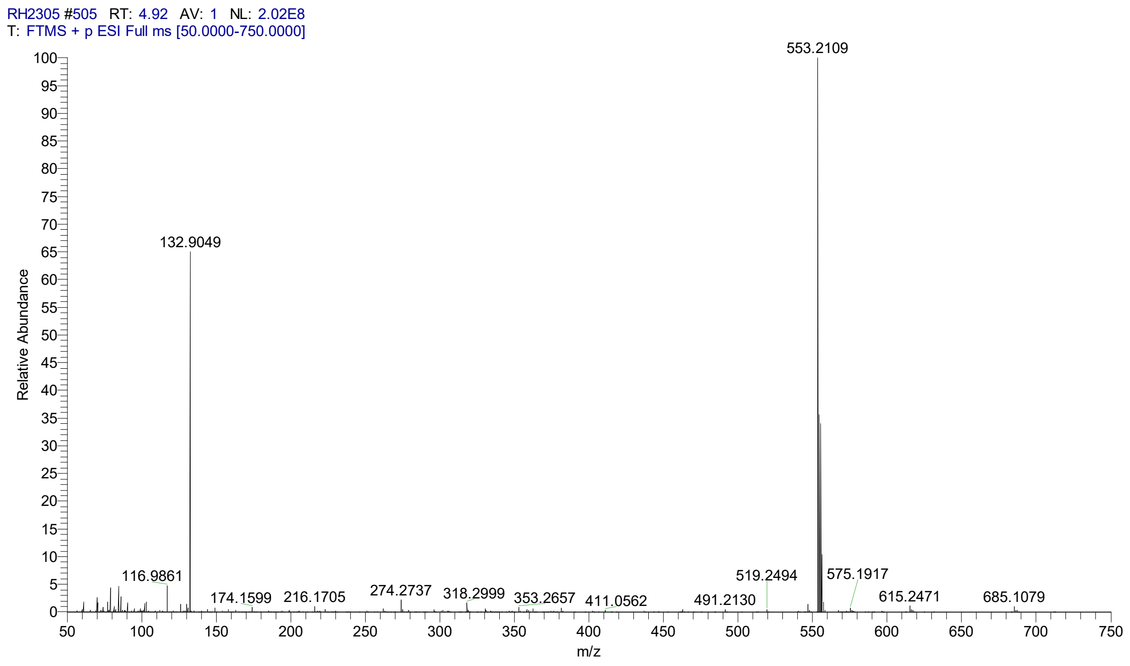

HR-MS Spectrum of compound **3o**
